# The Operationalisation of Sex and Gender in Quantitative Health–Related Research: A Scoping Review

**DOI:** 10.3390/ijerph19127493

**Published:** 2022-06-18

**Authors:** Sophie Horstmann, Corinna Schmechel, Kerstin Palm, Sabine Oertelt-Prigione, Gabriele Bolte

**Affiliations:** 1 Department of Social Epidemiology, Institute of Public Health and Nursing Research, University of Bremen, 28359 Bremen, Germany; gabriele.bolte@uni-bremen.de; 2 Health Sciences Bremen, University of Bremen, 28359 Bremen, Germany; 3 Gender and Science Research Unit, Institute of History, Humboldt-University of Berlin, 10099 Berlin, Germany; schmecco@hu-berlin.de (C.S.); kerstin.palm@hu-berlin.de (K.P.); 4 Department of Primary and Community Care, Radboud University Medical Center, 6500 HB Nijmegen, The Netherlands; sabine.oertelt-prigione@radboudumc.nl; 5 Sex- and Gender-Sensitive Medicine Unit, University of Bielefeld, 33615 Bielefeld, Germany

**Keywords:** sex, gender, intersectionality, scoping review, operationalisation, quantitative health research, epidemiology

## Abstract

Current trends in quantitative health research have highlighted the inadequacy of the usual operationalisation of sex and gender, resulting in a growing demand for more nuanced options. This scoping review provides an overview of recent instruments for the operationalisation of sex and gender in health-related research beyond a concept of mutually exclusive binary categories as male or masculine vs. female or feminine. Our search in three databases (Medline, Scopus and Web of Science) returned 9935 matches, of which 170 were included. From these, we identified 77 different instruments. The number and variety of instruments measuring sex and/or gender in quantitative health-related research increased over time. Most of these instruments were developed with a US-American student population. The majority of instruments focused on the assessment of gender based on a binary understanding, while sex or combinations of sex and gender were less frequently measured. Different populations may require the application of different instruments, and various research questions may ask for different dimensions of sex and gender to be studied. Despite the clear interest in the development of novel sex and/or gender instruments, future research needs to focus on new ways of operationalisation that account for their variability and multiple dimensions.

## 1. Introduction

Sex and gender dimensions are relevant to all areas of health sciences, and the request for their consideration in health–related research has been growing in recent years [1,2,3,4,5]. This is exemplified by an increasing demand by journals, policymakers and research funders for more systematic and comprehensive integration of sex and gender [6,7]. One such example is the EU research framework program Horizon Europe, which underscores the integration of the gender dimension into research as an important aspect of the European strategy to improve gender equality [8].

In current research practice, researchers generally ask survey participants about their sex and/or gender without further defining their underlying assumptions of these concepts. The possible answer options given are man/male or woman/female, which are usually conceptualised as mutually exclusive [9]. This operationalisation of sex or gender lags behind the current thinking of biological and social sciences, as both sex and gender are considered multi–layered, variable and non–binary [2,9]. Sex is generally operationalised through biological markers, such as primary and secondary sexual organs, chromosomes and hormone concentrations [8]. The term gender is used to describe social aspects and acts on an individual as well as on a structural and symbolic level [2]. From a recent developmental biology perspective, sex and gender are seen as entangled and interacting throughout human life [9,10,11,12]. Several researchers have recommended acknowledging this constant interaction by using the term ‘sex/gender’ [13,14]. Furthermore, sex/gender interacts with other categories of social inequality and power relations [2,10]. For this reason, researchers are advised to take other social categories into account when conducting sex/gender-related health research [10,15,16].

In the current research, the terms and the concepts of sex and gender are often confused or used inconsistently. One example is the often used Bem Sex Role Inventory (BSRI) which should, based on its measured content, actually be called a ‘gender’ role inventory [9]. Guidelines on the consideration of sex/gender in research recommend carefully reflecting on the aspects of sex/gender that are under discussion and precisely using the corresponding terminology [12,17].

The complexity of sex/gender requires a differentiated assessment, and there is a need for precise instruments grounded in theory for the measurement of sex/gender [18]. Therefore, researchers have developed new approaches to measure different dimensions of sex and/or gender in recent years [15,18,19,20]. One example is the so–called two–step approach [21,22]. This instrument measures both sex/gender assigned at birth and gender (in terms of current gender identity) in a way that enables the classification of cisgender, transgender and other gender–diverse respondents [23]. Other researchers have focused on the development of instruments that analyse the relevance of sex and/or gender based on existing data [20,24,25,26]. One example of this approach is the ‘gender index’ of Pelletier and colleagues, which was created from existing cohort data and classified participants on a one–dimensional scale from very feminine to very masculine. One of the elements of the ‘gender index’ is the BSRI [20], which is a questionnaire developed in 1974 that is still widely used in health–related research today [1,27,28]. The BSRI measures participants’ match with a defined set of personality traits to assess their degree of femininity, masculinity and androgyny [29].

Within the interdisciplinary research project DIVERGesTOOL, we are developing a toolbox for an adequate assessment of sex/gender variety in quantitative health research. As a first step, we set out to map and evaluate the existing literature about the operationalisation of sex and gender in health–related research. Previous work to map and evaluate existing literature has mostly focused on the operationalisation of gender [30,31] and not on providing a broad and systematic overview. With this scoping review, we are trying to fill this gap and encourage a discussion about the operationalisation of sex and/or gender that inspires researchers to experiment with different instruments. Additionally, we want to identify areas that require further focus in the future.

The objectives of this review are:

(1) To identify and characterise instruments used in current quantitative health–related research to measure sex and/or gender;
(2) To provide an overview of whether and how sex and/or gender is conceptualised within the identified instruments.

With the term ‘instrument’, we refer to all questionnaire items, scales, indices and tools that have been developed by one author or a research team with the intention of measuring one or multiple dimensions of sex and/or gender.

## 2. Materials and Methods

We conducted a scoping review following the PRISMA Extension for Scoping Reviews (PRISMA-ScR) [32]. Beyond the mere description of the identified sex and/or gender instruments, we also included a critical appraisal.

### 2.1. Search Strategy

In August 2020, we searched the three electronic databases MEDLINE (via OVID), Web of Science Core Collection and Scopus. The search strategies were drafted by an experienced librarian and further refined through team discussion. The final search syntax consisted of five different search strings applied to the title and keywords. The complete search strategy can be found in the supplement (Supplementary Table S1). Four search strings covered different search terms related to the concepts of sex/gender and instruments. A fifth search string comprised search terms related to intersectionality and instruments in a simplified version in order to identify those publications that did not contain terms related to sex/gender in title or keywords but that measured aspects of sex/gender. Search results were exported to and assessed with EndNote to facilitate the selection process and reviewer coordination. Duplicates were removed electronically and also manually by one researcher.

### 2.2. Eligibility Criteria

We restricted our search to publications in English. We included all original studies describing the development, application, comparison, validation or test of one or multiple instruments that measure one or various dimensions of sex and/or gender. Based on the limitations described in the introduction of this review, we wanted to identify instruments that go beyond the simple classification of participants into the mutually exclusive binary sex/gender categories ‘man/male’ and ‘woman/female’ either based on a single item question or the assumption of the researchers. Therefore, we only included publications that used instruments following a different approach. We focused on the operationalisation of sex/gender in health–related research. For our review, we followed the definition of health provided by the World Health Organisation (WHO) [33] and included publications that analyse any health outcomes or health–related factors. As we were only interested in the instruments currently used, we included papers published online or in print from January 2000 through to August 2020. Studies that do not focus on humans and studies describing instruments developed or used to measure sex and/or gender in children and adolescents under 18 years old were excluded.

### 2.3. Selection Process

First, two reviewers independently screened the titles and abstracts of all results of the literature search against eligibility criteria. Any disagreements about eligibility were resolved by discussion and consultation with a third reviewer, if necessary. In a second step, full texts of all potentially relevant articles were checked for eligibility by two independent reviewers. After reading the first 50 publications, the two reviewers met and discussed the results. The strategy was refined before continuing with the selection process. The final selections of the two reviewers were compared, and any disagreements were solved by discussion.

### 2.4. Data Extraction and Further Characterisation of the Instruments

For data extraction, we developed a data–charting form for use in Microsoft Excel. The extraction was carried out by one reviewer and then double–checked by a second independent reviewer. Disagreements were resolved by discussion. After extracting the first twenty included papers, reviewers met to confirm and adapt the extraction criteria. We extracted data on article characteristics (e.g., publication year, country of origin, and disciplinary field), details on the sex and/or gender instrument(s) applied (e.g., name and version) and further information about the instrument’s evaluation and reasons for their use. A final version of the charting form can be found in the supplement (Supplementary Table S2).

In addition, further information was collected for all identified sex and/or gender instruments. We collected data on the instruments’ aim, their structure (e.g., number of items and sub–scales), and the instruments’ development (e.g., country and year of origin and characteristics of the population that was used for the development or the first validation). If any information was not yet available in the included publications, further research was done. Any missing information was marked as such. The research was performed independently by two researchers, and disagreements were solved by consensus and discussion with other reviewers if needed.

### 2.5. Analysis

For every instrument retrieved through the search, we recorded the study population included at the time of development, as well as the country of origin and discipline of the corresponding author (please see Supplementary Table S2 for further detail). We counted the frequency of application of every instrument and reported if instruments were used multiple times or validated in different population samples.

We classified the instruments based on their approach and the dimensions of sex and/or gender that were assessed. For this purpose, we established five differentiations:

(a) We distinguished between those instruments that have to be employed during data collection and those applied for secondary analysis using already existing data.
(b) We categorised the instruments based on measured dimensions of sex and/or gender and their approach. Since the terms ‘sex’ and ‘gender’ are often confused with each other [11,17,34] and we wanted to achieve comparability, we did not rely on the terminology used in the included publications. Instead, we grouped the instruments according to their captured content. All instruments assessing biological dimensions (e.g., hormonal transition) were categorised as measuring sex, while those focusing on social dimensions (e.g., gender identity) were considered as ‘gender instruments’. Measurements assessing both social and biological dimensions were categorised as ‘sex+gender instruments’.
(c) We additionally classified the instruments into external and self-assessment instruments. Within the external assessment instruments, the participants agree with certain behaviours or traits, and an external person (e.g., the researcher) then assigns the participants to a sex and/or gender category based on these answers. In contrast to this, within the self-assessment instruments, the participants classify themselves into one or multiple sex and/or gender categories.
(d) We distinguished between instruments that are used to assign participants to one or multiple sex and/or gender categories or those that measure the participants’ conformity, degree of belonging to a sex and/or gender category or the expectations and stress participants experience connected to a determined sex and/or gender role.
(e) We differentiated whether the instruments work only with the categories male/female respectively masculinity/femininity (while also allowing different approaches, e.g., various grades of femininity/masculinity) or whether they propose other sex and/or gender categories (e.g., developing the category ‘androgyny’ based on certain grades of masculinity and femininity). All information used for these categorisations was taken from the information provided by the authors who developed the instruments. The categorisation was performed by two independent researchers, and any disagreements were solved by discussion.

To assess temporal developments or trends, we plotted the year of publication of the included studies. For comparison, we also plotted the number of all publications released in the databases Medline (via Ovid), Scopus and Web of Science from January 2000 to August 2020. To achieve comparability, the four distributions (including studies and total numbers of publications in each of the three databases) of publication frequency per year were standardised before comparison. Therefore, for every distribution, we calculated the difference between the observed number of publications and the mean value of publications per year and divided the result by the standard deviation.

We used a bar chart to visualise the date of development and application of the recorded instruments. Date of development was defined as the publication date of the original instrument, and application dates were based on subsequent publications making use of the instrument. Both distributions were categorised according to (a) whether the instruments measure gender, sex, sex + gender and (b) whether the publications are primary publications or secondary analyses. Instruments that measure gender were split into (a) external categorisation of the participant’s gender (by researchers) or (b) self–assignment by the participants.

Additionally, we assessed whether the instruments already contain further differentiations of sex and/or gender categories by an internal structuring through further social categories in line with the intracategorical complexity approach of McCall [35].

## 3. Results

### 3.1. Characteristics of Publications and Instruments

**Figure 1:**
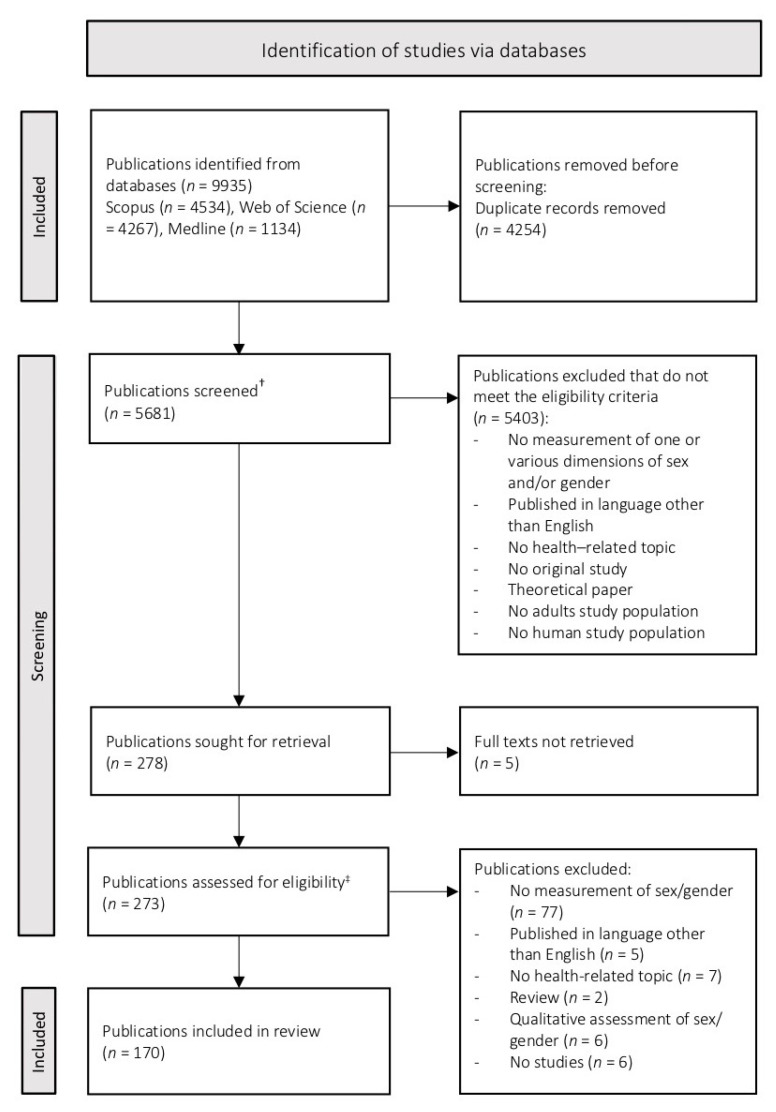
Flow diagram. † two reviewers independently screened title and abstracts, disagreements for 161 publications (2.8%), solved by discussion; ‡ two reviewers independently analysed the full articles, disagreements for 20 publications (7.3%), solved by discussion.

Our systematic search identified 9935 records (Figure 1). After the removal of duplicates, we screened 5681 potential eligible publications against eligibility criteria. Finally, we included 170 publications in the analysis of this review.

In total, we identified 77 different instruments for the quantitative assessment of sex and/or gender in quantitative health-related research that were applied in at least one of these 170 studies. Each instrument was given an individual ID number, consisting of the prefix “ID” and a number (e.g., ID1). For an overview of all instruments and the respective studies, please see Supplementary Table S3. Characteristics of the instruments that were identified within the included 170 articles are shown in Table 1.

In total, the instruments were applied 261 times within the 170 included publications. Most of the instruments have been used once or twice. However, the BSRI (ID20), including its short forms and translations, was used 59 times compared to 24 applications of the Conformity to Masculine Norms Inventory (CMNI, ID15), the second most frequently used instrument (Supplementary Figure S1).

Of the 77 instruments retrieved through the search, a huge amount has been developed with participants living in the USA (*n* = 33). Sorted by the study population, most of the instruments have been developed with populations that were either drawn from the general population (*n* = 13) or consisted of students or young adults only (*n* = 19). Ten of the latter instruments were developed with a population of students enrolled in a psychology program. Nineteen instruments were developed with single sex and/or gender populations consisting of either only men (*n* = 3), women (*n* = 6) or trans*people (*n* = 10). One instrument is an adaptation to adult populations (ID47) of an instrument originally developed for children.

Based on the publications within which the development of the instruments was described, most corresponding authors work in US-American research institutes (*n* = 34), and nine have been developed by Canadian research organisations. The majority of instruments have been developed in the field of psychology (*n* = 34), followed by public health (*n* = 9) and medicine (*n* = 8).

Most of the instruments (*n* = 14) have been developed with a rather small population of 100–500 participants (Supplementary Figure S2). The two most frequently employed instruments between January 2000 and August 2020, the BSRI (ID20) and CMNI (ID15), have been developed with populations of 194 and 752 participants, respectively.

### 3.2. Classification of Instruments

**Figure 2:**
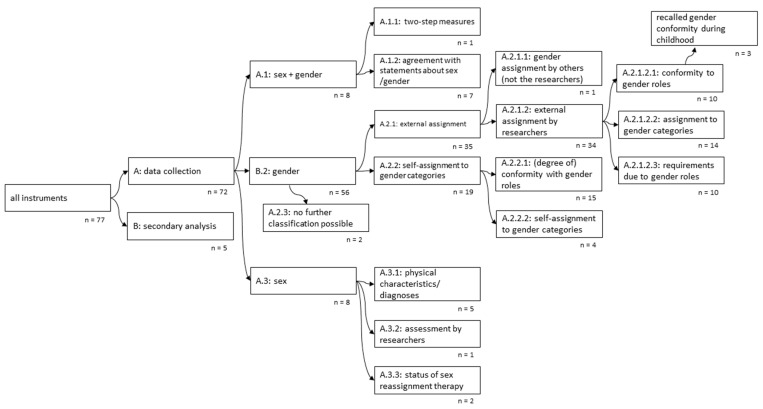
Tree diagram visualising the instruments’ methodology and measured dimensions of sex/gender.

The identified instruments were classified into different subgroups based on their methodology and the dimensions of sex and/or gender that were assessed (Figure 2).

The tree diagram illustrates how the identified 77 instruments can be split into increasingly specific groups based on the instruments’ approaches, including the assessed dimension(s) of sex and/or gender. Starting with the root node on the left, the instruments are split into those that have to be employed already for data collection (A) and those applied for secondary analysis (B) of existing data sets. The first group is split again based on the instruments’ ability to measure sex AND gender (A.1) or focus on either gender (A.2) OR sex (A.3). The instruments measuring sex and gender (A.1) are two–step measures (A.1.1) and instruments where participants assign their sex and/or gender on a physical and social level (A.1.2). Instruments assessing solely gender (A2) are split depending on whether they are based on an external assessment (A.2.1) or a self-assessment (A.2.2). Within the external assessment instruments, the participants agree with certain behaviours or traits. As these were previously linked to a gender category, the researchers or another person then subdivide the participants according to their answers. In contrast to this, within the self–assessment instruments, the participants classify themselves into one or multiple gender categories. Two instruments measuring gender did not enable this classification (A.2.3). The external–assignment instruments (A2.1) are split according to whether the assessment is made by persons close to the participants (A.2.2.1) or by the researchers (A.2.2.2). The former is divided into three subgroups: instruments which measure the participant’s conformity to a gender category (A.2.1.2.1), instruments which assign the participants to one or multiple gender categories (A.2.1.2.2), and instruments which measure the expectations and stress participant’s experience connected to a determined gender role (A.2.1.2.3). Instruments that are based on a self–assignment (A2.2) are split into those measuring participants’ self–assigned (degree of) gender conformity (A.2.2.1) and the self-assignment to gender categories (A.2.2.2). The last group of instruments are those that record sex based on physical characteristics (A.3). This group includes instruments that categorise participants on the basis of acquired data, e.g., DNA analyses or existing (intersex) diagnoses (A.3.1), sex assignment by researchers based on phenotype (A.3.2) and hormonal and surgical sex reassignment measures in a trans*population(A.3.3.).

The majority of instruments were used for data collection (*n* = 72). Most of these instruments (*n* = 56) are applied to exclusively assess gender. For the measurement of both dimensions (sex and gender) or exclusively sex, we identified eight instruments. The largest group of instruments focusing on the measurement of the participant’s gender are based on an external assessment by the researchers (*n* = 34). Five instruments are applicable for a secondary analysis of available data (see Supplementary Table S3 for the categorisation of the instruments).

### 3.3. Temporal Trends

**Figure 3:**
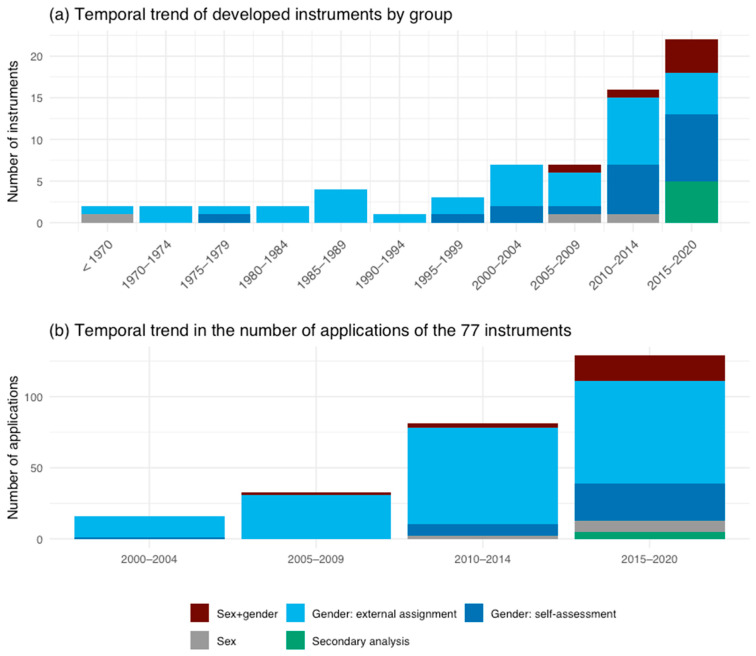
Temporal trends in instrument development and applications by group, one bar per 5-year period (**a**) temporal trends in developed instruments (*n* = 75, 2 instruments have to be excluded from this analysis due to missing information). (**b**) Temporal trends in applications (*n* = 261) of the 77 instruments identified in this review.

We could identify an increase in the number of instruments between 2000 and 2020 (Supplementary Figure S3). While in 2000, only three publications were identified, the number of relevant papers rose to 18 within the first 8 months of 2020. This observed trend is in line with the general increase in publications in the three databases Medline, Web of Science and Scopus within the last years. When looking at the time trends in instrument development (Figure 3a) and application (Figure 3b), an increase in the variability of instruments can be seen.

Before 2000, the development was almost exclusively focused on instruments measuring gender. From here on, a rise in the number of developed instruments measuring sex or combinations of sex and gender can be observed. The development of instruments for secondary analysis started in 2015.

Looking at the application of instruments, a similar picture can be observed. From 2000 to 2004, instruments that are based on an external assignment of gender account for almost all the instruments that have been applied within the included publications. Even though these instruments still account for the highest amount later, there was a steady rise in the number of applications of secondary analysis instruments, gender and/or sex assessment instruments, gender self–assignment instruments and sex assessment instruments from 2005 onwards. From 2015 to 2020, gender assessment instruments account for three–quarters of the instruments applied during this time period.

### 3.4. Classification of Instruments by Concepts of Sex/Gender

We identified 48 instruments in which sex/gender is conceptualised as male or masculine or female or feminine only.

Within these, a total of 26 instruments assign the participants to either a male or masculine or female or feminine sex or gender category. Out of these 26, 8 instruments do define maleness or masculinity and femaleness or femininity as mutually exclusive, while 18 measure independent scores of maleness or masculinity and femaleness or femininity.

Twenty-two instruments measure participants’ conformity (*n* = 14) or the consequences (gender role stress) of belonging to sex and/or gender (*n* = 8) in the categories of male or masculine or female or feminine only.

Twenty-five instruments were categorised as offering alternative answer options to male or masculine and female or feminine. Within these, fifteen instruments define participants belonging to a certain category, while eight instruments measure the conformity or level of belonging. Two instruments were categorised as mixed approaches because they first define the belonging to a certain sex and/or gender category before measuring the degree of belonging.

Two instruments had to be categorised as unclear since they do measure traits that are referred to as gender-related but do not use any gender categories; as such, they instead record levels of agency and communion (ID28) or factors such as marital status and household’s primary earner status (ID33).

The assignment of the identified instruments to the categories described above is shown in Supplementary Table S4.

### 3.5. Consideration of Other Social Categories

We identified 13 instruments that appear to directly consider other categories of social inequality and power relations while assessing sex and gender within the process of data collection. All of them work with the dimension of gender or sex + gender.

The main category of difference referred to is sexuality, which is reported in six of the instruments singularly and in one instrument in combination with other categories such as race, nationality and age (ID2). In practice, this included, e.g., instruments that view butches and femmes (two lesbian/female sexual minority identities) as valid gender categories within the category ‘female/feminine’ (ID43) or instruments which consider heterosexuality as an elementary part of stereotypical masculinity (e.g., ID15). Two instruments focus on the interdependency of gender on race, concretely with being Black (ID39 and ID40), and one instrument defines gender in accordance with culture/ethnicity and predefined age categories (ID30). Two instruments (ID36 and ID37) constitute a specific case, as they ask the participants to finish ten sentences starting with ‘As a woman/As a man…’. As the participants may or may not experience their gender in intersection with other social categories, it depends on their answers if the entanglement of sex and/or gender with other categories is considered within these instruments and which further categories of social inequality and power relations are taken into account. Of the thirteen instruments considering further social categories, nine originated in the field of psychology (Supplementary Figure S4).

## 4. Discussion

Our research demonstrates the availability of an increasing variety of instruments to measure sex and/or gender within the last 20 years. Most of these instruments have been developed with a rather restricted study population of US–American students and in the field of psychology. We could also identify different conceptualisations of sex and/or gender, ranging from mutually exclusive masculinity and femininity to multiple categories of sex and/or gender. The majority of instruments, however, measure gender based on a distinction between masculinity and femininity, although each was not considered as excluding the other.

The first aim of our review was the identification of instruments that are currently used in quantitative health research assessing one or multiple dimensions of sex and/or gender. Even though it was developed in 1974, the BSRI is still by far the most widely used instrument within the identified publications [1,27,28]. Although it is called the Sex Role Inventory, the instrument actually collects information about gender [9]. In the last decades, it has been increasingly criticised for building on and reproducing outdated gender stereotypes [1,27].

Like the BSRI, most instruments used for data collection were developed in the field of psychology and have been widely employed to assess the interrelation of different dimensions of gender and mental illnesses, such as eating disorders [36,37,38] and depression [39,40]. As the differentiated measure of sex/gender is not only relevant to psychological topics but to all kinds of biomedical and public health research, it should also be given a higher priority here [7]. On the contrary, the majority of the instruments applied for secondary analysis were developed in the fields of public health and medicine. This demonstrates that different research fields may require different kinds of instruments.

The majority of the identified instruments were developed in the USA. The development process often entailed asking a group of psychology students to rate social desirability to define categories of femininity and masculinity [29,41,42]. Given the homogeneity of the enrolled participants and their lack of representation of the general population, the developed instruments can exemplify a specific understanding of sex/gender [43]. For instance, attempts to reproduce Bem’s original desirability results with other populations led to differing outcomes [44,45]. Factor analyses could not confirm the underlying structures of the BSRI items either [46,47]. Overall, these findings raise questions about the transferability of instruments across different populations, especially when complex concepts such as sex/gender are being measured.

In line with a recently published scoping review by Miani and colleagues [30], we found that the majority of instruments are based on a binary distinction between femininity and masculinity, although, in many cases, they are not considered mutually exclusive. We identified several instruments that measure the participants’ degree of belonging to a sex and/or gender category or their conformity with stereotypical gender roles. A growing body of literature describes sex and/or gender as neither one-dimensional (femininity and masculinity as distinct ends of the sex and/or gender continuum) nor two-dimensional (femininity and masculinity as two separate dimensions) [48,49]. Rather, there can be multiple, possibly overlapping concepts of possibly overlapping masculinities and femininities as well within and across societies that should be taken into account [9,50,51].

A binary conceptualisation of sex and/or gender does not capture the broad variety [2] and excludes participants who identify outside the spectrum of femininity and masculinity [27]. Some of the identified instruments effectively move beyond this binary understanding [18,52] and are leading towards a more comprehensive view of sex and gender.

In general, the majority of identified instruments focus on the assessment of dimensions of gender. However, given the impact of biological sex on health and the entanglement of sex and gender [10,13,15], more comprehensive instruments focusing on the assessment of sex are needed. This requirement is partially met by the emergence of instruments measuring both sex and gender. These instruments allow the combined investigation of sex and gender within one instrument and offer an opportunity to represent their mutual influence [9]. Nevertheless, in most of these instruments, sex is defined as ‘sex assigned at birth’ [53] which is generally based on a visual assessment of the external genitalia at birth and influenced by cultural conventions [2]. This highlights the need for further expansion of the instruments to investigate sex as well.

Not every sex and/or gender dimension will be of interest for each health–related outcome. Rather, certain dimensions are of particular importance for individual research questions [54], and health-related outcomes can be influenced via different pathways [17]. Instruments to measure sex and/or gender might not be feasible in a one-size-fits-all format, and researchers should decide carefully which dimensions of sex and/or gender may be of interest to their specific research question [11,55]. Guidelines for the adequate consideration of sex and/or gender emphasize the need to consider categories and dimensions beyond a binary categorisation [9], and our current results demonstrate that this need is not met yet.

We could identify several instruments that operationalise gender or sex+gender as intertwined with other social categories. We only included instruments following an intercategorical approach [35]. Here, sex and/or gender are conceptualised as intersecting with other social positions and power relations and thus create further differentiation within the sex and/or gender categories. The most frequently included category is sexuality. Interestingly, sexuality was considered in differing ways within the different instruments. In 2016 the WHO defined the following three dimensions to define sexual orientation: sexual attraction/desire, sexual identity, and sexual behaviour [56]. When comparing, e.g., the ‘masculine’ CMNI (ID15) [57] with its ‘feminine’ version, the Conformity to Feminine Norms Inventory (CFNI) [41], the interaction of sexuality with stereotyping gender roles becomes evident. Both instruments reflect on participants´ sexuality as an intersecting part of gender conformity; however, the breadth of investigation differs. In fact, the CMNI considers all three WHO dimensions (in the items ‘Heterosexual Self-presentation’ and ‘Playboy’), while the female version, i.e., the CFNI, only includes sexual behaviour (in the items ‘Romantic relationship’ and ‘Sexual fidelity’).

Collecting information on sex/gender as intertwined with other social categories shows first considerations of the concept of intersectionality. The concept of intersectionality describes the interaction of distinct categories of social inequality and power relations to shape a person’s individual experiences and social positioning [10,58]. However, within our review, we only considered intracategorical approaches where sex and/or gender and other social categories were assessed within the same instrument. This approach needs to be distinguished from an independent collection of information on sex and/or gender and further categories of social inequality and power relations and an intracategorical assessment in the step of data analysis [59].

We could identify a small number of instruments that do not apply the terminology of femininity and masculinity. Instead, they measure gender–related factors or traits, such as levels of agency and communion (ID28) [60], or characteristics of social relations and socioeconomic position, such as marital status and household’s primary earner status (ID33) [37]. Nielsen and colleagues [31] recently developed a comparable approach with an instrument that measures gender–related behaviours and attitudes without a normative assignment to femininity or masculinity. This is also in line with the recommendations of Schellenberg and Kaiser, who encourage researchers to focus on the aspects of sex/gender they are interested in instead of relying on proxy terms [61]. Such an approach would no longer focus on the categorisation of participants into sex and/or gender categories but on the analysis of possible mechanisms [59,62]. Hence, these instruments could capture gender-related factors relevant for health-related research without reproducing essentialist binary sex/gender stereotypes. At the same time, they present the opportunity to take into account the structural level of sex/gender by considering different pathways and mechanisms.

This review has some limitations. We conducted our search in three databases and limited it to publications in English, which could explain the dominance of US-based, Western approaches in the identified literature. For future research, it might be interesting to include a wider range of languages and sources to create the possibility of identifying a greater variety of different instruments. Since we could not rely on the sex and/or gender terminology used in the publications, we had to classify the instruments based on our own assumptions, which might have caused some misclassification. As it led to such a large number of hits that would not have been able to be processed, we had to exclude the terms ‘men’ and ‘women’ from our search strategy. This might have resulted in an underrepresentation of instruments measuring sex.

A major strength of this review is the interdisciplinary composition of the DIVERGesTOOL study group, comprising expertise in gender studies, epidemiology, public health and gender medicine, which enabled us to include different perspectives in the whole research process. Furthermore, we conducted a thorough and comprehensive search following the PRISMA Extension for Scoping Reviews, which allowed a broad range of approaches to measuring sex and/or gender to be included within our scoping review. This distinguishes our review from previous reviews that focused on gender only [30,31]. Thus, our scoping review clearly demonstrates that further elaboration or new development of instruments to operationalise sex and gender comprehensively are needed to be able to assess the impact of sex/gender dimensions on health and to fulfil the requirements of expert associations, funding agencies, and scientific journals to consider sex and gender in research.

## 5. Conclusions

Our review identified an increasing variety of instruments for the operationalisation of sex and/or gender in quantitative health–related research over time. However, most of these instruments operationalise sex and/or gender in the context of a mostly binary representation of masculinity and femininity. Different instruments might be needed to investigate sex and/or gender in diverse populations and to address different research questions. There is a clear interest in and need for the development of novel instruments to measure sex and/or gender in more comprehensive terms in the field of health–related research. Two major future challenges result from our overview. First, the domain of biological sex needs more expansive instruments to capture its variations. It has to be clarified to which extent this is feasible in data collection by interview or requires physiological and laboratory measurements. Second, a fundamental discussion about the advantages of gender classification in masculinity/femininity terms versus its operationalisation as traits and behaviours embedded in a societal context is needed. The ambivalence to balance at this point is whether the labelling of certain traits or behaviours as masculine or feminine is reproducing binary gender stereotypes. Nevertheless, the query of participants’ attitudes and conformity to common gender roles creates the possibility of taking the social context and power relations into account when analysing sex/gender. Thus, the consideration of gender roles might be of relevance in certain health-related contexts and in the disclosure of different gendered pathways leading to health-related outcomes. Addressing both challenges in future research will clearly benefit the awareness of the relevance of sex and gender for health-related research.

## Figures and Tables

**Table 1:** Characteristics of the instruments retrieved through the search (*n* = 77).

Characteristics		* n *	%
Country of corresponding author ^†^	USA	34	53.1
	Canada	9	14.1
	Australia	3	4.7
	Germany	3	4.7
	Netherlands	3	4.7
	Austria	2	3.1
	United Kingdom	2	3.1
	Finland	1	1.6
	India	1	1.6
	Italy	1	1.6
	Japan	1	1.6
	New Zealand	1	1.6
	Poland	1	1.6
	Sweden	1	1.6
	Switzerland	1	1.6
	*Not specified*	13	
Discipline of corresponding author ^†^	Psychology	38	61.3
	Public health	9	14.5
	Medicine	8	12.9
	Sociology	2	3.2
	Interdisciplinary network	2	3.2
	Communication science	1	1.6
	Neuroscience	1	1.6
	Nursing	1	1.6
	*Not specified*	15	
Characteristics of study population of instrument development	Students General population	17 13	28.3 21.7
	Trans* people	10	16.7
	Patients with certain mental or physical health conditions (and controls)	8	13.
	Women only	6	10.0
	Men only	3	5.
	Young adult	2	3.3
	Children	1	1.7
	*Not specified*	17	
Country of study population of instrument development	USA	34	55.7
	Canada	6	9.8
	Several countries	5	8.2
	United Kingdom	3	4.9
	Netherlands	3	4.9
	Australia	2	3.3
	Finland	1	1.6
	Germany	1	1.6
	India	1	1.6
	Italy	1	1.6
	Japan	1	1.6
	Singapore	1	1.6
	Sweden	1	1.6
	Switzerland	1	1.6
	*Not specified*	16	

† Based on the publication within which the development of the instrument is described (please see Supplementary Table S2 for details).

## Data Availability

Not applicable.

## Supplementary Materials

The following supporting information can be downloaded at: https://www.mdpi.com/article/10.3390/ijerph19127493/s1, Table S1: Search algorithm; Table S2: Extracted variables and additionally collected information; Table S3: Instruments, Figure S1: Number of applications, Figure S2: Population size, Figure S3: Temporal trends of the included publications, Table S4: Categorisation of the tools according to their underlying concepts of sex and/or gender, Figure S4: Consideration of further social categories. References [63,64,65,66,67,68,69,70,71,72,73,74,75,76,77,78,79,80,81,82,83,84,85,86,87,88,89,90,91,92,93,94,95,96,97,98,99,100,101,102,103,104,105,106,107,108,109,110,111,112,113,114,115,116,117,118,119,120,121,122,123,124,125,126,127,128,129,130,131,132,133,134,135,136,137,138,139,140,141,142,143,144,145,146,147,148,149,150,151,152,153,154,155,156,157,158,159,160,161,162,163,164,165,166,167,168,169,170,171,172,173,174,175,176,177,178,179,180,181,182,183,184,185,186,187,188,189,190,191,192,193,194,195,196,197,198,199,200,201,202,203,204,205,206,207,208,209,210,211,212,213,214,215,216,217,218,219,220,221,222,223] are cited in the supplementary materials.
