# Supplementary material for: The Operationalisation of Sex and Gender in Quantitative Health–Related Research: A Scoping Review
Source: Int J Environ Res Public Health. 2022 Jun 18;19(12):7493. doi: 10.3390/ijerph19127493 (PMC9224188; doi:10.3390/ijerph19127493)
Supplement: Supplementary file 1 [file ijerph-19-07493-s001.zip › ijerph-1680960-supplementary.pdf]

Supplementary material: The operationalisation of sex and gender in quantitative health-related research: a scoping review

Sophie Horstmann [1,2,*], Corinna Schmechel [3], Kerstin Palm [3], Sabine Oertelt-Prigione [4,5] and Gabriele Bolte [1,2]

[1] University of Bremen, Institute of Public Health and Nursing Research, Department of Social Epidemiology, Bremen, Germany; gabriele.bolte@uni-bremen.de (GB)
[2] Health Sciences Bremen, University of Bremen, Bremen, Germany
[3] Gender and Science Research Unit, Institute of History, Humboldt-University of Berlin, Germany; schmecco@uni-bremen.de (CS); kerstin.palm@hu-berlin.de (K.P.)
[4] Department of Primary and Community Care, Radboud University Medical Center, Nijmegen, Netherlands; sabine.oertelt-prigione@radboudumc.nl (SOP)
[5] Sex- and Gender-Sensitive Medicine Unit, University of Bielefeld, Bielefeld, Germany

* Correspondence: sophie.horstmann@uni-bremen.de (SH)

Supplementary Table S1: Search algorithm

**Search term 1:**	((Gender or sex) adj3 (index or indices or score* or measure* or scale* or operational* or survey* or questionnaire* or inventor* or guideline* or tool* or framework* or approach or method*))
**Search term 2:**	((transgender or transsex* or intersex*) adj3 (index or indices or score* or measure* or scale* or operational* or survey* or questionnaire* or inventor* or guideline* or tool* or framework* or approach or method*))
**Search term 3:**	((feminin* or masculin*) adj3 (index or indices or score* or measure* or scale* or operational* or survey* or questionnaire* or inventor* or guideline* or tool* or framework* or approach or method*))
**Search term 4:**	(Androgyny adj3 (index or indices or score* or measure* or scale* or operational* or survey* or questionnaire* or inventor* or guideline* or tool* or framework* or approach or method*))
**Search term 5:**	(intersection* adj3 (index or indices or score* or measure* or scale* or operational* or survey* or questionnaire* or inventor* or guideline* or tool* or framework* or approach or method*))

Title- and keyword search in Medline, Scopus and Web of Science
All five search terms were connected with an "or".
Adj3: the two search terms are only allowed to be three words apart from each other in any order

Supplementary Table S2: Extracted variables and additionally collected information

Extracted Variable	Definition
Author(s)	Author(s) of the publication
Year	Date the publication was released
Title	Title of the publication
Country	Country the publication was written, determined by the institutional affiliation of the corresponding author
Aim of the Study	Objectives of the study, determined by the information in the publication
Discipline	Discipline/ research field of the publication, determined by the institutional affiliation of the corresponding author
Study population	Characteristics and size of the population(s) with which the described study was conducted
Name of instrument	Name of the instrument that was applied within the publication to assess sex and/or gender
Version of the applied instrument	If applicable the instrument's version was determined by the information in the publication
Aim of the instrument	The aim of the instrument/ the measured sex/gender related content was determined by the information in the publication
Evaluation of the instrument	Findings of validations that were conducted within the publication

Additionally collected information

Collected Information	Definition
Name of instrument	Name of the instrument
Existing versions of instrument	If possible, all existing versions of the instrument were collected
Original author(s)	Author(s) who developed or first validated the instrument
Year of origin	Year the publication describing the development of the instrument was published
Country of origin	Country where the instrument was developed, determined by the institutional affiliation of the corresponding author of the publication where the development was described
Discipline of origin	Discipline/ research field of the publication describing the development of the instrument, determined by the institutional affiliation of the corresponding author
Aim of the scale	The aim of the instrument/ the measured sex/gender related content
Data collection or secondary analysis	We distinguished between those that have to be employed during data collection and those applied for secondary analysis
Population of origin	Population with which the instrument was developed or first validated
Measurement of sex and/or gender	All instruments assessing biological dimensions (e.g., hormonal transition) were categorised as measuring sex, while those focusing on social dimensions (e.g., gender identity) were considered as "gender instruments". measurements assessing both, social and biological dimensions, were categorised as "sex+gender instruments"
Type and mode	We assessed the type of instrument (e.g., item(s), scale) and mode (e.g., questionnaire, interview)
Different versions of the instrument	We assessed if there are different versions of the instrument for different sex and/or gender groups
Consideration of other social categories	The instruments already contain further differentiations of sex and/or gender by an internal structuring through further social categories

Supplementary Table S3: Instruments

ID	Instrument	Author and date of the publication describing the instruments' development	Author and date of publications included within the critical review
**A.1: Assessment of gender and sex**			
**ID1**	Two-step measures	Various sources	Bauer et al., 2017 [1]<br>Cella et al., 2013 [2]<br>Hart et al., 2019 [3]<br>Jones et al., 2019 [4]<br>Lombardi and Banik, 2016 [5]<br>McGuire et al., 2019 [6]<br>Michaels et al., 2017 [7]<br>Nagata et al., 2020 [8]<br>Pickett et al., 2020 [9]<br>Reisner et al., 2014a [10]<br>Reisner et al., 2014b [11]<br>Tamar-Mattis et al., 2018 [12]<br>Tate et al., 2015 [13]<br>Taube and Mussap, 2020 [14]<br>Walls et al., 2019 [15]
**A.1.2: Sex and/or gender traits measure**			
**ID2**	Perceived Masculinity Questionnaire	Chesebro and Fuse, 2001 [16]	Chesebro and Fuse, 2001 [16]
**ID3**	Multidimensional sex/gender items	Bauer, 2012 [17]	Bauer et al., 2017 [1]
**ID4**	Multidimensional Sex/Gender Measure (MSGM)	Bauer et al., 2017 [1]	Bauer et al., 2017 [1]
**ID5**	Gender Preoccupation and Stability Questionnaire (GPSQ)	Hakeem et al., 2016 [18]	Hakeem et al., 2016 [18]
**ID6**	Assessment of (trans)gender transition	Jones et al., 2019 [4]	Jones et al., 2019 [4]
**ID7**	Assessment of (trans)gender spectrum	Scheim and Bauer, 2015[†] [19]	Dharma et al., 2019 [20]
**ID8**	Gender Identity Scale (GIS)	Ho and Mussap, 2019 [21]	Ho and Mussap, 2019 [21]<br>Ho and Mussap, 2020 [22]

**A.2.1.1: Gender assignment by others (not the researchers)**			
**ID9**	Scale for a close friend to rate participant's masculinity/ femininity	Pletzer et al., 2015 [23]	Gruber et al., 2020 [24]
**A.2.1.2.1: Conformity to one preassigned gender role**			
**ID10**	Recalled childhood gender nonconformity	Zucker et al., 2006 [25]	Green et al., 2018 [26]<br>Reisner et al., 2014a [10]<br>Singh et al., 2010 [27]<br>Veale, 2016 [28]<br>Zucker et al., 2006 [25]
**ID11**	Childhood Gender Nonconformity Scale	Bailey and Oberschneider, 1997† [29]	Skidmore et al., 2006 [30]
**ID12**	Childhood gender role behaviour (CGRB)	Kachel et al., 2016 [31]	Kachel et al., 2016 [31]
**ID13**	Auburn Differential Masculinity Inventory	Burk et al., 2004 [32]	Burk et al., 2004 [32]
**ID14**	Masculine Gender Identity in Females	Blanchard and Freund, 1983† [33]	Lehavot et al., 2011 [34]

**ID15**	Conformity to Masculine Norms Inventory (CMNI)	Mahalik et al., 2003† [35]	Alt et al., 2014 [36]<br>Brabete and Sánchez-López, 2012 [37]<br>Brabete et al., 2013 [38]<br>Burn and Ward, 2005 [39]<br>Easton, 2014 [40]<br>Esteban-Gonzalo et al., 2019 [41]<br>Hammer et al., 2018a [42]<br>Hayley et al., 2017 [43]<br>Levant et al., 2015 [44]<br>Levant et al., 2020 [45]<br>Ludlow and Mahalik, 2001 [46]<br>Magovcevic and Addis, 2008 [47]<br>Murray et al., 2013 [48]<br>Owen, 2011 [49]<br>Parent and Moradi, 2009 [50]<br>Parent and Moradi, 2011 [51]<br>Parent and Smiler, 2013 [52]<br>Rice et al., 2013 [53]<br>Rochelle and Yim, 2015 [54]<br>Steinfeldt et al., 2011 [55]<br>Wide et al., 2011 [56]<br>Wong et al., 2013 [57]
**ID16**	Conformity to Feminine Norms Inventory (CFNI)	Mahalik et al., 2005† [58]	Aparicio-García and Alvarado-Izquierdo, 2019 [59]<br>Brabete and Sánchez-López, 2012 [37]<br>Brabete et al., 2013 [38]<br>Brown et al., 2020 [60]<br>Esteban-Gonzalo et al., 2019 [41]<br>Green et al., 2008 [61]<br>Hayley et al., 2017 [43]<br>Kling et al., 2017 [62]<br>Lyocsa and Lyocsa, 2013 [63]<br>Mahalik et al., 2005 [58]<br>Murray et al., 2013 [48]<br>Parent and Moradi, 2010 [64]<br>Parent and Moradi, 2011 [65]

**ID17**	Gender Identity/Gender Dysphoria Questionnaire for Adolescents and Adults (GIGDQ-AA)	Deogracias et al., 2007 [66]	Deogracias et al., 2007 [66]<br>Fisher et al., 2017 [67]<br>Galupo and Pulice-Farrow, 2020 [68]<br>George and Stokes, 2018 [69]<br>Haghighat et al., 2019 [70]<br>Hakeem et al., 2016 [18]<br>Schneider et al., 2016 [71]<br>Singh et al., 2010 [27]<br>Taskinen et al., 2016 [72]
**ID18**	Utrecht Gender Dysphoria Scale (UGDS)	Cohen-Kettenis and Van Goozen, 1997† [73]	Galupo and Pulice-Farrow, 2020 [68]<br>Jürgensen et al., 2013 [74]<br>Kreukels et al., 2018 [75]<br>Schneider et al., 2016 [71]<br>van de Grift et al., 2017 [76]
**ID19**	Socially assigned gender nonconformity	Wylie et al., 2010† [77]	Reisner et al., 2014a [10]<br>Reisner et al., 2014b [11]
**A.2.1.2.2: Assignment to one (of several) gender category by researchers**			
**ID20**	Bem Sex Role Inventory (BSRI)	Bem, 1974† [78]	Choi et al., 2007 [79]<br>Choi et al., 2008 [80]<br>Choi et al., 2009 [81]<br>Costa et al., 2019 [82]<br>Crosby and Sprock, 2004 [83]<br>Endut et al., 2020 [84]<br>Factor and Rothblum, 2017 [85]<br>Ferrer-Pérez and Bosch-Fiol, 2014 [86]<br>Fisher et al., 2010 [87]<br>Geldenhuys and Bosch, 2020 [88]<br>Ghiasİ, 2019 [89]<br>Gómez-Gil et al., 2012 [90]<br>Gruber et al., 2020 [24]<br>Haghighat et al., 2019 [70]<br>Hepp et al., 2005 [91]<br>Hoffman and Borders, 2001 [92]<br>Hunt et al., 2007 [93]

			Johnson et al., 2006 [94]<br>Juster and Lupien, 2012 [95]<br>Kachel et al., 2016 [31]<br>Kamboj et al., 2014 [96]<br>Karim et al., 2019 [97]<br>Klingemann and Gomez, 2010 [98]<br>Kurpisz et al., 2016 [99]<br>Lehavot et al., 2011 [34]<br>Mahalik et al., 2005 [58]<br>March et al., 2013 [100]<br>Nielsen et al., 2015 [101]<br>Norris et al., 2017 [102]<br>O'Kelly, 2011 [103]<br>Oswald, 2004 [104]<br>Özkan and Lajunen, 2005 [105]<br>Pelletier et al., 2015 [106]<br>Peng, 2006 [107]<br>Peralta et al., 2010 [108]<br>Raparelli et al., 2020 [109]<br>Reynolds et al., 2016 [110]<br>Ritter, 2004 [111]<br>Russell and Keel, 2002 [112]<br>Sajatovic et al., 2011 [113]<br>Stafford et al., 2000 [114]<br>Tardif and Van Gijseghem, 2005 [115]<br>Taskinen et al., 2016 [72]<br>Tate et al., 2013 [116]<br>Tate, 2011 [117]<br>Vafaei et al., 2014 [118]<br>Vafaei et al., 2016 [119]<br>van Well et al., 2005 [120]<br>Zhang et al., 2001 [121]
**ID21**	Minnesota Multiphasic Personality Inventory (MMPI)	Hathaway & McKinley 1937‡	Taher, 2007 [122]<br>Tardif and Van Gijseghem, 2005 [115]

**ID22**	Minnesota Multiphasic Personality Inventory 2 MMPI-2	Butcher et al. 1989‡	Alavi et al., 2015 [123]<br>Haghighat et al., 2019 [70]<br>Karim et al., 2019 [97]<br>Park et al., 2020 [124]<br>Woo and Oei, 2006 [125]<br>Woo and Oei, 2008 [126]
**ID23**	Personal Attributes Questionnaire (PAQ)	Helmreich et al. 1974‡	Gruber et al., 2020 [24]<br>Kachel et al., 2016 [31]<br>Langelier et al., 2018 [127]<br>Lehavot et al., 2011 [34]<br>Luyt, 2018 [128]<br>Peralta et al., 2010 [108]<br>van Well et al., 2005 [120]
**ID24**	Psychological Gender Inventory	Kuczyńska 1992‡	Kowalczyk et al., 2012 [129]<br>Mandal and Kocur, 2013 [130]
**ID25**	SEX Role Behaviour Scale (SRBS)	(Orlofsky, 1981)†	McCreary et al., 2002 [131]
**ID26**	Positive–Negative Sex-Role Inventory (PN-SRI)	(Berger and Krahé, 2013)†	Rydberg Sterner et al., 2018 [132]
**ID27**	Masculinity - Humanity - Femininity (MHF) scale	Ito 1978‡	Hirokawa et al., 2001 [133]
**ID28**	Gender Role Inventory	Weaver and Sargent, 2007 [134]	Weaver and Sargent, 2007 [134]
**ID29**	Singapore Androgyny Inventory	Ward, 2000 [135]	Ward, 2000 [135]
**ID30**	Indian Gender Role Identity Scale (IGRIS)	Basu, 2010† [136]	Basu et al., 2012 [137]
**ID31**	Gender-Related Attributes Survey	Gruber et al., 2020 [24]	Gruber et al., 2020 [24]
**ID32**	Gender role behaviour scale	Athenstaedt, 2000[31]	Kachel et al., 2016
**ID33**	Assessment of gender related factors	Raparelli et al., 2020 [109]	Raparelli et al., 2020 [109]
**A.2.1.2.3: Requirements due to gender role**			

**ID34**	Masculine Gender Role Stress	Eisler and Skidmore, 1987† [138]	Arrindell et al., 2003 [139]<br>Kaźmierczak et al., 2013 [140]<br>Klingemann and Gomez, 2010 [98]<br>Mussap, 2008 [141]<br>Susánszky and Döbrössy, 2019 [142]<br>Swartout et al., 2015 [143]<br>van Well et al., 2005 [120]<br>Wong et al., 2013 [57]
**ID35**	Feminine Gender Role Stress	Gillespie and Eisler, 1992† [144]	Kaźmierczak et al., 2013 [140]<br>Richmond et al., 2015 [145]<br>van Well et al., 2005 [120]
**ID36**	Subjective Femininity Stress Scale	Shea et al., 2014 [146]	Shea et al., 2014 [146]
**ID37**	Subjective Masculinity Stress Scale (SMSS)	Wong et al., 2013 [57]	Wong et al., 2013 [57]
**ID38**	Gender Role Conflict Scale (GRCS)	O'Neil et al., 1986† [147]	Alt et al., 2014 [36]<br>Hammer et al., 2018b [148]<br>Herdman et al., 2012 [149]<br>Jones and Heesacker, 2012 [150]<br>Komlenac et al., 2018 [151]<br>Lease et al., 2009 [152]<br>Levant et al., 2015 [44]<br>Luyt, 2018 [128]<br>Moradi et al., 2000 [153]<br>Norwalk et al., 2011 [154]<br>Wester et al., 2012 [155]<br>Wong et al., 2013 [57]<br>Zhang et al., 2015 [156]
**ID39**	Masculinity Inventory Scale (MIS) for Black Men	Mincey et al., 2014 [157]	Mincey et al., 2014 [157]
**ID40**	Gendered Racial-Ethnic Socialization Scale for Black Women	Brown et al., 2017 [158]	Brown et al., 2017 [158]
**ID41**	O'Kelly Women's Belief Scales	O'Kelly, 2011 [103]	O'Kelly, 2011 [103]
**ID42**	Masculine Self-esteem Scale (MSES)	Clark et al., 2003 [159]	Langelier et al., 2018 [127]

**ID43**	Gender Expression Measure among Sexual Minority Women (GEM-SMW)	Lehavot et al., 2011 [34]	Lehavot et al., 2011 [34]
**A.2.2.1: Agreement with statements on conformity with one's own gender role or assessment of the degree of one's own conformity (not category)**			
**ID44**	Transgender Congruence scale	Kozee et al., 2012 [160]	Jones et al., 2019 [4]<br>Kozee et al., 2012 [160]<br>Taube and Mussap, 2020 [14]
**ID45**	Strength of Transgender Identity Scale	Barr et al., 2016† [161]	Taube and Mussap, 2020 [14]
**ID46**	Genderqueer Identity (GQI) Scale	McGuire et al., 2019 [6]	McGuire et al., 2019 [6]
**ID47**	Measure of adult gender typicality	Egan and Perry, 2001 [162]	Tate et al., 2015 [13]
**ID48**	Gender nonconformity in self-presentation (of gay men)	Reisen et al., 2013 [163]	Reisen et al., 2013 [163]
**ID49**	Transgender Behaviour Scale	Docter and Fleming, 2001 [164]	Bockting et al., 2020 [165]<br>Docter and Fleming, 2001 [164]
**ID50**	Traditional Masculinity-Femininity (TMF) scale	Kachel et al., 2016 [31]	Kachel et al., 2016 [31]
**ID51**	Gender Expression Identity	Lehavot et al., 2011 [34]	Lehavot et al., 2011 [34]
**ID52**	Bipolar gender identity continuum	Tate, 2011 [117]	Tate, 2011 [117]
**ID53**	Scale of perceived femininity and masculinity	Taskinen et al., 2016 [72]	Taskinen et al., 2016 [72]
**ID54**	Sex-Role Identity Scale (SRIS)	Storms, 1979 [166]	Lehavot et al., 2011 [34]
**ID55**	Measure of Gender Perception	Bejerot and Eriksson, 2014 [167]	Bejerot and Eriksson, 2014 [167]
**ID56**	Six item sex role scale	Pletzer et al., 2015† [23]	Gruber et al., 2020 [24]
**ID57**	Self-rated gender scales	Hart et al., 2019 [3]	Hart et al., 2019 [3]
**ID58**	Reference Group Identity Dependence Scale (RGIDS)	Wade and Gelso, 1998† [168]	Alt et al., 2014 [36]
**A.2.2.2: Self-assignment to gender categories**			
**ID59**	Single item assessing transgender-identity	Bockting et al., 2020 [165]	Bockting et al., 2020 [165]

**ID60**	Self-identification as transgender	Information not available	Cicero et al., 2020 [169]<br>Levant et al., 2020 [45]
**ID61**	Three questions form transgender survey	Factor and Rothblum, 2017 [85]	Factor and Rothblum, 2017 [85]
**ID62**	Single item measure assessing gender identity	Various sources	Bauerband et al., 2019 [170]<br>Bazzi et al., 2015 [171]<br>Christian et al., 2018 [172]<br>Fraser et al., 2020 [173]<br>George and Stokes, 2018 [69]<br>Ho and Mussap, 2020 [22]<br>Kozee et al., 2012 [160]<br>Kreukels et al., 2018 [75]<br>Pickett et al., 2020 [9]
**A.2.3: No further classification possible**			
**ID63**	Measurement of Masculinity-Femininity [MF]	Information not available	Ewalds-Kvist et al., 2003 [174]
**ID64**	Questionnaire of Gender Identity	Information not available	Jürgensen et al., 2013 [74]
**A.3: Assessment of Sex**			
**A.3.1: Measurement of physical characteristics/ diagnoses**			
**ID65**	DNA analysis	Various sources	Ballering et al., 2020 [175]
**ID66**	Examination of internal and external genital organs	Various sources	Ballering et al., 2020 [175]
**ID67**	Androgyny Score	Tanner, 1951† [176]	Dubey, 2015 [177]<br>Mishra et al., 2011 [178]
**ID68**	Assessment of karyotype and androgen effect	Various sources	(Kreukels et al., 2018)
**ID69**	Single item measure about DSD condition	The GenIUSS Group., 2014† [179]	Tamar-Mattis et al., 2018 [12]
**A.3.2: Assessment by researchers**			
**ID70**	Physical Appearance Scale	Smith et al., 2005† [180]	van de Grift et al., 2017 [76]
**A.3.3: Status of sex reassignment therapy**			

**ID71**	Status of gender affirming hormone therapy	Various sources	Suppakitjanusant et al., 2020 [181]
**ID72**	Status of sex reassignment surgery	Various sources	(Cella et al., 2013)
**B: Secondary analysis**			
**ID73**	Gender Index	Ballering et al., 2020 [175]	Ballering et al., 2020 [175]
**ID74**	Gender Index	Norris et al., 2017 [102]	Norris et al., 2017 [102]
**ID75**	GENDER Index	Lacasse et al., 2020 [182]	Lacasse et al., 2020 [182]
**ID76**	Gender Score	Pelletier et al., 2015 [106]	Pelletier et al., 2015 [106]
**ID77**	Labour Force Gender Index (LFGI)	Smith and Koehoorn, 2016 [183]	Smith and Koehoorn, 2016 [183]

Subtitles according to categorization in figure 2; †publications that were additionally researched but were not part of the original search; ‡publications of which full texts could not be retrieved

References

1. Bauer, G.R.; Braimoh, J.; Scheim, A.I.; Dharma, C. Transgender-Inclusive Measures of Sex/Gender for Population Surveys: Mixed-Methods Evaluation and Recommendations. *PLoS ONE* **2017**, *12*, e0178043, doi:10.1371/journal.pone.0178043.
2. Cella, S.; Iannaccone, M.; Cotrufo, P. Influence of Gender Role Orientation (Masculinity versus Femininity) on Body Satisfaction and Eating Attitudes in Homosexuals, Heterosexuals and Transsexuals. *Eat Weight Disord* **2013**, *18*, 115–124, doi:10.1007/s40519-013-0017-z.
3. Hart, C.G.; Saperstein, A.; Magliozzi, D.; Westbrook, L. Gender and Health: Beyond Binary Categorical Measurement. *J Health Soc Behav* **2019**, *60*, 101–118, doi:10.1177/0022146519825749.
4. Jones, B.A.; Bouman, W.P.; Haycraft, E.; Arcelus, J. The Gender Congruence and Life Satisfaction Scale (GCLS): Development and Validation of a Scale to Measure Outcomes from Transgender Health Services. *International Journal of Transgenderism* **2019**, *20*, 63–80, doi:10.1080/15532739.2018.1453425.
5. Lombardi, E.; Banik, S. The Utility of the Two-Step Gender Measure Within Trans and Cis Populations. *Sex Res Soc Policy* **2016**, *13*, 288–296, doi:10.1007/s13178-016-0220-6.
6. McGuire, J.K.; Beek, T.F.; Catalpa, J.M.; Steensma, T.D. The Genderqueer Identity (GQI) Scale: Measurement and Validation of Four Distinct Subscales with Trans and LGBQ Clinical and Community Samples in Two Countries. *International Journal of Transgenderism* **2019**, *20*, 289–304, doi:10.1080/15532739.2018.1460735.
7. Michaels, S.; Milesi, C.; Stern, M.; Viox, M.H.; Morrison, H.; Guerino, P.; Dragon, C.N.; Haffer, S.C. Improving Measures of Sexual and Gender Identity in English and Spanish to Identify LGBT Older Adults in Surveys. *LGBT Health* **2017**, *4*, 412–418, doi:10.1089/lgbt.2016.0168.
8. Nagata, J.M.; Murray, S.B.; Compte, E.J.; Pak, E.H.; Schauer, R.; Flentje, A.; Capriotti, M.R.; Lubensky, M.E.; Lunn, M.R.; Obedin-Maliver, J. Community Norms for the Eating Disorder Examination Questionnaire (EDE-Q) among Transgender Men and Women. *Eating Behaviors* **2020**, *37*, 101381, doi:10.1016/j.eatbeh.2020.101381.
9. Pickett, A.C.; Valdez, D.; Barry, A.E. Measurement Implications Associated with Refinement of Sexual and Gender Identity Survey Items: A Case Study of the National College Health Assessment. *Journal of American College Health* **2020**, *68*, 720–726, doi:10.1080/07448481.2019.1598421.
10. Reisner, S.L.; Conron, K.J.; Tardiff, L.A.; Jarvi, S.; Gordon, A.R.; Austin, S.B. Monitoring the Health of Transgender and Other Gender Minority Populations: Validity of Natal Sex and Gender Identity Survey Items in a U.S. National Cohort of Young Adults. *BMC Public Health* **2014**, *14*, 1224, doi:10.1186/1471-2458-14-1224.
11. Reisner, S.L.; Biello, K.; Rosenberger, J.G.; Austin, S.B.; Haneuse, S.; Perez-Brumer, A.; Novak, D.S.; Mimiaga, M.J. Using a Two-Step Method to Measure Transgender Identity in Latin America/the Caribbean, Portugal, and Spain. *Arch Sex Behav* **2014**, *43*, 1503–1514, doi:10.1007/s10508-014-0314-2.
12. Tamar-Mattis, S.; Gamarel, K.E.; Kantor, A.; Baratz, A.; Tamar-Mattis, A.; Operario, D. Identifying and Counting Individuals with Differences of Sex Development Conditions in Population Health Research. *LGBT Health* **2018**, *5*, 320–324, doi:10.1089/lgbt.2017.0180.
13. Tate, C.C.; Bettergarcia, J.N.; Brent, L.M. Re-Assessing the Role of Gender-Related Cognitions for Self-Esteem: The Importance of Gender Typicality for Cisgender Adults. *Sex Roles* **2015**, *72*, 221–236, doi:10.1007/s11199-015-0458-0.
14. Taube, L.N.; Mussap, A.J. Evaluating the Transgender Positive Identity Measure (T-PIM) across Intersecting Identities. *Journal of Gay & Lesbian Mental Health* **2020**, *25*, 20–53, doi:10.1080/19359705.2020.1789019.
15. Walls, N.E.; Kattari, S.K.; Speer, S.R.; Kinney, M.K. Transfeminine Spectrum Parenting: Evidence from the National Transgender Discrimination Survey. *Social Work Research* **2019**, *43*, 133–144, doi:10.1093/swr/svz005.
16. Chesebro, J.W.; Fuse, K. The Development of a Perceived Masculinity Scale. *Communication Quarterly* **2001**, *49*, 203–278, doi:10.1080/01463370109385628.
17. Bauer, G. Making Sure Everyone Counts: Considerations for Inclusion, Identification and Analysis of Transgender and Transsexual Participants in Health Surveys / Pour s'assurer Que Tout Le Monde Compte : Facteurs Pouvant Justifier l'inclusion, l'identification et l'analyse de Participants Transgenres et Transsexuels Dans Les Enquêtes Sur La Santé. **2012**, doi:10.14288/1.0132676.
18. Hakeem, A.; Črnčec, R.; Asghari-Fard, M.; Harte, F.; Eapen, V. Development and Validation of a Measure for Assessing Gender Dysphoria in Adults: The Gender Preoccupation and Stability Questionnaire. *International Journal of Transgenderism* **2016**, *17*, 131–140, doi:10.1080/15532739.2016.1217812.

19. Scheim, A.I.; Bauer, G.R. Sex and Gender Diversity Among Transgender Persons in Ontario, Canada: Results From a Respondent-Driven Sampling Survey. *The Journal of Sex Research* **2015**, *52*, 1–14, doi:10.1080/00224499.2014.893553.
20. Dharma, C.; Scheim, A.I.; Bauer, G.R. Exploratory Factor Analysis of Two Sexual Health Scales for Transgender People: Trans-Specific Condom/Barrier Negotiation Self-Efficacy (T-Barrier) and Trans-Specific Sexual Body Image Worries (T-Worries). *Arch Sex Behav* **2019**, *48*, 1563–1572, doi:10.1007/s10508-018-1383-4.
21. Ho, F.; Mussap, A.J. The Gender Identity Scale: Adapting the Gender Unicorn to Measure Gender Identity. *Psychology of Sexual Orientation and Gender Diversity* **2019**, *6*, 217–231, doi:10.1037/sgd0000322.
22. Ho, F.; Mussap, A.J. Development of the Trans and Gender Diverse Social Anxiety Scale. *Anxiety, Stress, & Coping* **2020**, *33*, 675–697, doi:10.1080/10615806.2020.1768533.
23. Pletzer, B.; Petasis, O.; Ortner, T.M.; Cahill, L. Interactive Effects of Culture and Sex Hormones on the Sex Role Self-Concept. *Front. Neurosci.* **2015**, *9*, doi:10.3389/fnins.2015.00240.
24. Gruber, F.M.; Distlberger, E.; Scherndl, T.; Ortner, T.M.; Pletzer, B. Psychometric Properties of the Multifaceted Gender-Related Attributes Survey (GERAS). *European Journal of Psychological Assessment* **2020**, *36*, 612–623, doi:10.1027/1015-5759/a000528.
25. Zucker, K.J.; Mitchell, J.N.; Bradley, S.J.; Tkachuk, J.; Cantor, J.M.; Allin, S.M. The Recalled Childhood Gender Identity/Gender Role Questionnaire: Psychometric Properties. *Sex Roles* **2006**, *54*, 469–483, doi:10.1007/s11199-006-9019-x.
26. Green, L.; Rimes, K.A.; Rahman, Q. Beliefs About Others' Perceptions—Gender Typicality: Scale Development and Relationships to Gender Nonconformity, Sexual Orientation, and Well-Being. *The Journal of Sex Research* **2018**, *55*, 837–849, doi:10.1080/00224499.2017.1384785.
27. Singh, D.; Deogracias, J.J.; Johnson, L.L.; Bradley, S.J.; Kibblewhite, S.J.; Owen-Anderson, A.; Peterson-Badali, M.; Meyer-Bahlburg, H.F.L.; Zucker, K.J. The Gender Identity/Gender Dysphoria Questionnaire for Adolescents and Adults: Further Validity Evidence. *Journal of Sex Research* **2010**, *47*, 49–58, doi:10.1080/00224490902898728.
28. Veale, J.F. Factorial Validity and Invariance Assessment of a Short Version of the Recalled Childhood Gender Identity/Role Questionnaire. *Arch Sex Behav* **2016**, *45*, 537–550, doi:10.1007/s10508-015-0684-0.
29. Bailey, J.M.; Oberschneider, M. Sexual Orientation and Professional Dance. *Archives of Sexual Behavior volume* **1997**, *26*, 433–444.
30. Skidmore, W.C.; Linsenmeier, J.A.W.; Bailey, J.M. Gender Nonconformity and Psychological Distress in Lesbians and Gay Men. *Arch Sex Behav* **2006**, *35*, 685–697, doi:10.1007/s10508-006-9108-5.
31. Kachel, S.; Steffens, M.C.; Niedlich, C. Traditional Masculinity and Femininity: Validation of a New Scale Assessing Gender Roles. *Front. Psychol.* **2016**, *7*, doi:10.3389/fpsyg.2016.00956.
32. Burk, L.R.; Burkhart, B.R.; Sikorski, J.F. Construction and Preliminary Validation of the Auburn Differential Masculinity Inventory. *Psychology of Men & Masculinity* **2004**, *5*, 4–17, doi:10.1037/1524-9220.5.1.4.
33. Blanchard, R.; Freund, K. Measuring Masculine Gender Identity in Females. *Journal of Consulting and Clinical Psychology* **1983**, *51*, 205–214, doi:10.1037/0022-006X.51.2.205.
34. Lehavot, K.; King, K.M.; Simoni, J.M. Development and Validation of a Gender Expression Measure Among Sexual Minority Women. *Psychology of Women Quarterly* **2011**, *35*, 381–400, doi:10.1177/0361684311413554.
35. Mahalik, J.R.; Locke, B.D.; Ludlow, L.H.; Diemer, M.A.; Scott, R.P.J.; Gottfried, M.; Freitas, G. Development of the Conformity to Masculine Norms Inventory. *Psychology of Men & Masculinity* **2003**, *4*, 3–25, doi:10.1037/1524-9220.4.1.3.
36. Alt, M.; Lewis, A.M.; Liu, W.M.; Vilain, E.; Sánchez, F.J. On the Validity of Popular Masculinity Rating Scales with Gay Men. *Arch Sex Behav* **2014**, *43*, 1547–1557, doi:10.1007/s10508-014-0363-6.
37. Brabete, A.C.; Sánchez-López, M. del P. How Does the Gender Influence People's Health? Data of a Sample of Romanian People Living in Spain. *Procedia - Social and Behavioral Sciences* **2012**, *33*, 148–152, doi:10.1016/j.sbspro.2012.01.101.
38. Brabete, A.C.; Sánchez-López, M. del P.; Cuéllar-Flores, I.; Rivas-Diez, R. The Impact of Gender Norms on Alcohol and Tobacco Use at Romanians. *Procedia - Social and Behavioral Sciences* **2013**, *78*, 230–234, doi:10.1016/j.sbspro.2013.04.285.
39. Burn, S.M.; Ward, A.Z. Men's Conformity to Traditional Masculinity and Relationship Satisfaction. *Psychology of Men & Masculinity* **2005**, *6*, 254–263, doi:10.1037/1524-9220.6.4.254.
40. Easton, S.D. Masculine Norms, Disclosure, and Childhood Adversities Predict Long-Term Mental Distress among Men with Histories of Child Sexual Abuse. *Child Abuse & Neglect* **2014**, *38*, 243–251, doi:10.1016/j.chiabu.2013.08.020.

41. Esteban-Gonzalo, L.; Manso-Martínez, M.E.; Botín-González, P.; Manchado-Simal, B.; Rodrigo-de-Frutos, R.M.; González-Pascual, J.L. The Relationship between Conformity to Male and Female Gender Norms and Depression during Pregnancy. *Arch Womens Ment Health* **2019**, *22*, 809–815, doi:10.1007/s00737-019-01003-0.
42. Hammer, J.H.; Heath, P.J.; Vogel, D.L. Fate of the Total Score: Dimensionality of the Conformity to Masculine Norms Inventory-46 (CMNI-46). *Psychology of Men & Masculinity* **2018**, *19*, 645–651, doi:10.1037/men0000147.
43. Hayley, A.; Cox, E.; Zinkiewicz, L.; Graham, K.; Wells, S.; Zhou, J.; Miller, P.G. Barroom Aggression Perpetration by Australian Women: Associations with Heavy Episodic Drinking, Trait Aggression, and Conformity to Gender Norms. *Journal of Substance Use* **2017**, *22*, 597–604, doi:10.1080/14659891.2016.1271040.
44. Levant, R.F.; Hall, R.J.; Weigold, I.K.; McCurdy, E.R. Construct Distinctiveness and Variance Composition of Multi-Dimensional Instruments: Three Short-Form Masculinity Measures. *Journal of Counseling Psychology* **2015**, *62*, 488–502, doi:10.1037/cou0000092.
45. Levant, R.F.; McDermott, R.; Parent, M.C.; Alshabani, N.; Mahalik, J.R.; Hammer, J.H. Development and Evaluation of a New Short Form of the Conformity to Masculine Norms Inventory (CMNI-30). *Journal of Counseling Psychology* **2020**, *67*, 622–636, doi:10.1037/cou0000414.
46. Ludlow, L.H.; Mahalik, J.R. Congruence between a Theoretical Continuum of Masculinity and the Rasch Model: Examining the Conformity to Masculine Norms Inventory. *J Appl Meas* **2001**, *2*, 205–226.
47. Magovcevic, M.; Addis, M.E. The Masculine Depression Scale: Development and Psychometric Evaluation. *Psychology of Men & Masculinity* **2008**, *9*, 117–132, doi:10.1037/1524-9220.9.3.117.
48. Murray, S.B.; Rieger, E.; Karlov, L.; Touyz, S.W. Masculinity and Femininity in the Divergence of Male Body Image Concerns. *J Eat Disord* **2013**, *1*, 11, doi:10.1186/2050-2974-1-11.
49. Owen, J. Assessing the Factor Structures of the 55- and 22-Item Versions of the Conformity to Masculine Norms Inventory. *Am J Mens Health* **2011**, *5*, 118–128, doi:10.1177/1557988310363817.
50. Parent, M.C.; Moradi, B. Confirmatory Factor Analysis of the Conformity to Masculine Norms Inventory and Development of the Conformity to Masculine Norms Inventory-46. *Psychology of Men & Masculinity* **2009**, *10*, 175–189, doi:10.1037/a0015481.
51. Parent, M.C.; Davis-Delano, L.R.; Morgan, E.M.; Woznicki, N.W.; Denson, A. An Inductive Analysis of Young Adults' Conceptions of Femininity and Masculinity and Comparison to Established Gender Inventories. *Gend. Issues* **2020**, *37*, 1–24, doi:10.1007/s12147-019-09246-y.
52. Parent, M.C.; Smiler, A.P. Metric Invariance of the Conformity to Masculine Norms Inventory-46 among Women and Men. *Psychology of Men & Masculinity* **2013**, *14*, 324–328, doi:10.1037/a0027642.
53. Rice, S.M.; Fallon, B.J.; Aucote, H.M.; Möller-Leimkühler, A.M. Development and Preliminary Validation of the Male Depression Risk Scale: Furthering the Assessment of Depression in Men. *Journal of Affective Disorders* **2013**, *151*, 950–958, doi:10.1016/j.jad.2013.08.013.
54. Rochelle, T.L.; Yim, K.H. Assessing the Factor Structure of the Chinese Conformity to Masculine Norms Inventory. *The Journal of Psychology* **2015**, *149*, 29–41, doi:10.1080/00223980.2013.837023.
55. Steinfeldt, J.A.; Gilchrist, G.A.; Halterman, A.W.; Gomory, A.; Steinfeldt, M.C. Drive for Muscularity and Conformity to Masculine Norms among College Football Players. *Psychology of Men & Masculinity* **2011**, *12*, 324–338, doi:10.1037/a0024839.
56. Wide, J.; Mok, H.; McKenna, M.; Ogrodniczuk, J.S. Effect of Gender Socialization on the Presentation of Depression among Men: A Pilot Study. *Can Fam Physician* **2011**, *57*, e74-78.
57. Wong, Y.J.; Shea, M.; Hickman, S.J.; LaFollette, J.R.; Cruz, N.; Boghokian, T. The Subjective Masculinity Stress Scale: Scale Development and Psychometric Properties. *Psychology of Men & Masculinity* **2013**, *14*, 148–155, doi:10.1037/a0027521.
58. Mahalik, J.R.; Morray, E.B.; Coonerty-Femiano, A.; Ludlow, L.H.; Slattery, S.M.; Smiler, A. Development of the Conformity to Feminine Norms Inventory. *Sex Roles* **2005**, *52*, 417–435, doi:10.1007/s11199-005-3709-7.
59. Aparicio-García, M.E.; Alvarado-Izquierdo, J.M. Is There a "Conformity to Feminine Norms" Construct? A Bifactor Analysis of Two Short Versions of Conformity to Feminine Norms Inventory. *Curr Psychol* **2019**, *38*, 1110–1120, doi:10.1007/s12144-018-9815-9.
60. Brown, E.L.; Ovrebo, E.; Emery, H.E.; Stenersen, M. The Validity of the Conformity to Feminine Norms Inventory with Sexual and Gender Minority Women. *Journal of LGBT Issues in Counseling* **2020**, *14*, 56–68, doi:10.1080/15538605.2020.1711289.
61. Green, M.A.; Davids, C.M.; Skaggs, A.K.; Riopel, C.M.; Hallengren, J.J. Femininity and Eating Disorders. *Eating Disorders* **2008**, *16*, 283–293, doi:10.1080/10640260802115829.

62. Kling, J.; Holmqvist Gattario, K.; Frisén, A. Swedish Women's Perceptions of and Conformity to Feminine Norms. *Scand J Psychol* **2017**, *58*, 238–248, doi:10.1111/sjop.12361.
63. Lyocsa, I.; Lyocsa, S. Confirmatory Factor Analysis of the Abbreviated Conformity to Feminine Norms Inventory. *Social Work Research* **2013**, *37*, 414–422, doi:10.1093/swr/svt034.
64. Parent, M.C.; Moradi, B. Confirmatory Factor Analysis of the Conformity to Feminine Norms Inventory and Development of an Abbreviated Version: The CFNI-45. *Psychology of Women Quarterly* **2010**, *34*, 97–109, doi:10.1111/j.1471-6402.2009.01545.x.
65. Parent, M.C.; Moradi, B. An Abbreviated Tool for Assessing Feminine Norm Conformity: Psychometric Properties of the Conformity to Feminine Norms Inventory–45. *Psychological Assessment* **2011**, *23*, 958–969, doi:10.1037/a0024082.
66. Deogracias, J.J.; Johnson, L.L.; Meyer-Bahlburg, H.F.L.; Kessler, S.J.; Schober, J.M.; Zucker, K.J. The Gender Identity/Gender Dysphoria Questionnaire for Adolescents and Adults. *Journal of Sex Research* **2007**, *44*, 370–379, doi:10.1080/00224490701586730.
67. Fisher, A.D.; Castellini, G.; Ristori, J.; Casale, H.; Giovanardi, G.; Carone, N.; Fanni, E.; Mosconi, M.; Ciocca, G.; Jannini, E.A.; et al. Who Has the Worst Attitudes toward Sexual Minorities? Comparison of Transphobia and Homophobia Levels in Gender Dysphoric Individuals, the General Population and Health Care Providers. *J Endocrinol Invest* **2017**, *40*, 263–273, doi:10.1007/s40618-016-0552-3.
68. Galupo, M.P.; Pulice-Farrow, L. Subjective Ratings of Gender Dysphoria Scales by Transgender Individuals. *Arch Sex Behav* **2020**, *49*, 479–488, doi:10.1007/s10508-019-01556-2.
69. George, R.; Stokes, M.A. Gender Identity and Sexual Orientation in Autism Spectrum Disorder. *Autism* **2018**, *22*, 970–982, doi:10.1177/1362361317714587.
70. Haghighat, F.; Shirazi, E.; Ardebili, M.E.; Alavi, K. Psychometric Assessment of the Persian Version of a Dimensional Instrument to Measure Gender Identity Disorder. *Latinoamericana de Hipertensión* **2019**, *14*, 346–353.
71. Schneider, C.; Cerwenka, S.; Nieder, T.O.; Briken, P.; Cohen-Kettenis, P.T.; De Cuypere, G.; Haraldsen, I.R.; Kreukels, B.P.C.; Richter-Appelt, H. Measuring Gender Dysphoria: A Multicenter Examination and Comparison of the Utrecht Gender Dysphoria Scale and the Gender Identity/Gender Dysphoria Questionnaire for Adolescents and Adults. *Arch Sex Behav* **2016**, *45*, 551–558, doi:10.1007/s10508-016-0702-x.
72. Taskinen, S.; Suominen, J.S.; Mattila, A.K. Gender Identity and Sex Role of Patients Operated on for Bladder Exstrophy-Epispadias. *Journal of Urology* **2016**, *196*, 531–535, doi:10.1016/j.juro.2016.02.2961.
73. Cohen-Kettenis, P.T.; Van Goozen, S.H.M. Sex Reassignment of Adolescent Transsexuals: A Follow-up Study. *Journal of the American Academy of Child & Adolescent Psychiatry* **1997**, *36*, 263–271, doi:10.1097/00004583-199702000-00017.
74. Jürgensen, M.; Kleinemeier, E.; Lux, A.; Steensma, T.D.; Cohen-Kettenis, P.T.; Hiort, O.; Thyen, U.; Köhler, B. Psychosexual Development in Adolescents and Adults with Disorders of Sex Development—Results from the German Clinical Evaluation Study. *The Journal of Sexual Medicine* **2013**, *10*, 2703–2714, doi:10.1111/j.1743-6109.2012.02751.x.
75. Kreukels, B.P.C.; Köhler, B.; Nordenström, A.; Roehle, R.; Thyen, U.; Bouvattier, C.; de Vries, A.L.C.; Cohen-Kettenis, P.T.; Köhler, B.; Cohen-Kettenis, P.; et al. Gender Dysphoria and Gender Change in Disorders of Sex Development/Intersex Conditions: Results From the Dsd-LIFE Study. *The Journal of Sexual Medicine* **2018**, *15*, 777–785, doi:10.1016/j.jsxm.2018.02.021.
76. van de Grift, T.C.; Elaut, E.; Cerwenka, S.C.; Cohen-Kettenis, P.T.; De Cuypere, G.; Richter-Appelt, H.; Kreukels, B.P.C. Effects of Medical Interventions on Gender Dysphoria and Body Image: A Follow-Up Study. *Psychosom Med* **2017**, *79*, 815–823, doi:10.1097/PSY.0000000000000465.
77. Wylie, S.A.; Corliss, H.L.; Boulanger, V.; Prokop, L.A.; Austin, S.B. Socially Assigned Gender Nonconformity: A Brief Measure for Use in Surveillance and Investigation of Health Disparities. *Sex Roles* **2010**, *63*, 264–276, doi:10.1007/s11199-010-9798-y.
78. Bem, S.L. The Measurement of Psychological Androgyny. *Journal of Consulting and Clinical Psychology* **1974**, *42*, 155–162, doi:10.1037/h0036215.
79. Choi, N.; Fuqua, D.R.; Newman, J.L. Hierarchical Confirmatory Factor Analysis of the Bem Sex Role Inventory. *Educational and Psychological Measurement* **2007**, *67*, 818–832, doi:10.1177/0013164406299106.
80. Choi, N.; Fuqua, D.R.; Newman, J.L. The Bem Sex-Role Inventory: Continuing Theoretical Problems. *Educational and Psychological Measurement* **2008**, *68*, 881–900, doi:10.1177/0013164408315267.
81. Choi, N.; Fuqua, D.R.; Newman, J.L. Exploratory and Confirmatory Studies of the Structure of the Bem Sex Role Inventory Short Form With Two Divergent Samples. *Educational and Psychological Measurement* **2009**, *69*, 696–705, doi:10.1177/0013164409332218.

82. Costa, P.A.; Garcia, I.Q.; Pimenta, F.; Marôco, J.; Leal, I. Late-Onset Hypogonadism (LOH), Masculinity and Relationship and Sexual Satisfaction: Are Sexual Symptoms of LOH Mediators of Traditional Masculinity on Relationship and Sexual Satisfaction? *Sex. Health* **2019**, *16*, 389, doi:10.1071/SH18165.
83. Crosby, J.P.; Sprock, J. Effect of Patient Sex, Clinician Sex, and Sex Role on the Diagnosis of Antisocial Personality Disorder: Models of Underpathologizing and Overpathologizing Biases. *J. Clin. Psychol.* **2004**, *60*, 583–604, doi:10.1002/jclp.10235.
84. Endut, N.; Bagheri, R.; Azman, A.; Hashim, I.H.M.; Selamat, N.H.; Mohajer, L. The Effect of Gender Role on Attitudes Towards Inequitable Gender Norms Among Malaysian Men. *Sexuality & Culture* **2020**, *24*, 2113–2136, doi:10.1007/s12119-020-09740-6.
85. Factor, R.J.; Rothblum, E.D. A Comparison of Trans Women, Trans Men, Genderqueer Individuals, and Cisgender Brothers and Sisters on the Bem Sex-Role Inventory: Ratings by Self and Siblings. *Journal of Homosexuality* **2017**, *64*, 1872–1889, doi:10.1080/00918369.2016.1273717.
86. Ferrer-Pérez, V.A.; Bosch-Fiol, E. The Measure of the Masculinity–Femininity Construct Today: Some Reflections on the Case of the Bem Sex Role Inventory / La Medida Del Constructo Masculinidad–Feminidad En La Actualidad: Algunas Reflexiones Sobre El Caso Del Bem Sex Role Inventory. *Revista de Psicología Social* **2014**, *29*, 180–207, doi:10.1080/02134748.2013.878569.
87. Fisher, A.D.; Bandini, E.; Ricca, V.; Ferruccio, N.; Corona, G.; Meriggiola, M.C.; Jannini, E.A.; Manieri, C.; Ristori, J.; Forti, G.; et al. Dimensional Profiles of Male to Female Gender Identity Disorder: An Exploratory Research. *The Journal of Sexual Medicine* **2010**, *7*, 2487–2498, doi:10.1111/j.1743-6109.2009.01687.x.
88. Geldenhuys, M.; Bosch, A. A Rasch Adapted Version of the 30-Item Bem Sex Role Inventory (BSRI). *Journal of Personality Assessment* **2020**, *102*, 428–439, doi:10.1080/00223891.2018.1527343.
89. Ghiasİ, A. The Effect of Gender-Role Orientation on Attitudes towards Menstruation in a Sample of Female University Students. *J Turkish German Gynecol Assoc* **2019**, *20*, 138–141, doi:10.4274/jtgga.galenos.2018.2018.0122.
90. Gómez-Gil, E.; Gómez, A.; Cañizares, S.; Guillamón, A.; Rametti, G.; Esteva, I.; Vázquez, A.; Salamero-Baró, M. Clinical Utility of the Bem Sex Role Inventory (BSRI) in the Spanish Transsexual and Nontranssexual Population. *Journal of Personality Assessment* **2012**, *94*, 304–309, doi:10.1080/00223891.2011.650302.
91. Hepp, U.; Spindler, A.; Milos, G. Eating Disorder Symptomatology and Gender Role Orientation. *Int. J. Eat. Disord.* **2005**, *37*, 227–233, doi:10.1002/eat.20087.
92. Hoffmann, W.; Latza, U.; Baumeister, S.E.; Brünger, M.; Buttmann-Schweiger, N.; Hardt, J.; Hoffmann, V.; Karch, A.; Richter, A.; Schmidt, C.O.; et al. Guidelines and Recommendations for Ensuring Good Epidemiological Practice (GEP): A Guideline Developed by the German Society for Epidemiology. *Eur J Epidemiol* **2019**, *34*, 301–317, doi:10.1007/s10654-019-00500-x.
93. Hunt, K.; Lewars, H.; Emslie, C.; Batty, G.D. Decreased Risk of Death from Coronary Heart Disease amongst Men with Higher "femininity" Scores: A General Population Cohort Study. *International Journal of Epidemiology* **2007**, *36*, 612–620, doi:10.1093/ije/dym022.
94. Johnson, H.D.; Mcnair, R.; Vojick, A.; Congdon, D.; Monacelli, J.; Lamont, J. CATEGORICAL AND CONTINUOUS MEASUREMENT OF SEX-ROLE ORIENTATION: DIFFERENCES IN ASSOCIATIONS WITH YOUNG ADULTS'REPORTS OF WELL-BEING. *soc behav pers* **2006**, *34*, 59–76, doi:10.2224/sbp.2006.34.1.59.
95. Juster, R.-P.; Lupien, S. A Sex- and Gender-Based Analysis of Allostatic Load and Physical Complaints. *Gender Medicine* **2012**, *9*, 511–523, doi:10.1016/j.genm.2012.10.008.
96. Kamboj, S.K.; Oldfield, L.; Loewenberger, A.; Das, R.K.; Bisby, J.; Brewin, C.R. Voluntary and Involuntary Emotional Memory Following an Analogue Traumatic Stressor: The Differential Effects of Communality in Men and Women. *Journal of Behavior Therapy and Experimental Psychiatry* **2014**, *45*, 421–426, doi:10.1016/j.jbtep.2014.05.001.
97. Karim, H.; Shirazi, E.; Nohesara, S.; Sadeghi, H.M.; Saeb, A.; Alavi, K. Comparison of Gender Roles in Male and Female in Patients with Borderline Personality Disorder (BPD) with Control Group and It's Correlation with Severity of Clinical Symptoms. *Revista Latinoamericana de Hipertens* **2019**, *14*, 1–7.
98. Klingemann, H.; Gomez, V. Masculinity Issues in Addiction Treatment in Swiss Inpatient Alcohol Programs: Bringing Men's Treatment Needs Back to the Research Agenda. *Journal of Men's Health* **2010**, *7*, 211–220, doi:10.1016/j.jomh.2010.04.003.
99. Kurpisz, J.; Mak, M.; Lew-Starowicz, M.; Nowosielski, K.; Bieńkowski, P.; Kowalczyk, R.; Misiak, B.; Frydecka, D.; Samochowiec, J. Personality Traits, Gender Roles and Sexual Behaviours of Young Adult Males. *Ann Gen Psychiatry* **2016**, *15*, 28, doi:10.1186/s12991-016-0114-2.

100. March, E.; Grieve, R.; Marx, E.; Witteveen, K. More of a (Wo)Man Offline? Gender Roles Measured in Online and Offline Environments. *Personality and Individual Differences* **2013**, *55*, 887–891, doi:10.1016/j.paid.2013.07.018.
101. Nielsen, K.J.; Hansen, C.D.; Bloksgaard, L.; Christensen, A.-D.; Jensen, S.Q.; Kyed, M. The Impact of Masculinity on Safety Oversights, Safety Priority and Safety Violations in Two Male-Dominated Occupations. *Safety Science* **2015**, *76*, 82–89, doi:10.1016/j.ssci.2015.02.021.
102. Norris, C.M.; Johnson, N.L.; Hardwicke-Brown, E.; McEwan, M.; Pelletier, R.; Pilote, L. The Contribution of Gender to Apparent Sex Differences in Health Status Among Patients with Coronary Artery Disease. *Journal of Women's Health* **2017**, *26*, 50–57, doi:10.1089/jwh.2016.5744.
103. O'Kelly, M. Psychometric Properties of the O'Kelly Women's Belief Scales. *J Rat-Emo Cognitive-Behav Ther* **2011**, *29*, 145–157, doi:10.1007/s10942-010-0120-2.
104. Oswald, P.A. An Examination of the Current Usefulness of the Bem Sex-Role Inventory. *Psychol Rep* **2004**, *94*, 1331–1336, doi:10.2466/pr0.94.3c.1331-1336.
105. Özkan, T.; Lajunen, T. Masculinity, Femininity, and the Bem Sex Role Inventory in Turkey. *Sex Roles* **2005**, *52*, 103–110, doi:10.1007/s11199-005-1197-4.
106. Pelletier, R.; Ditto, B.; Pilote, L. A Composite Measure of Gender and Its Association With Risk Factors in Patients With Premature Acute Coronary Syndrome. *Psychosomatic Medicine* **2015**, *77*, 517–526, doi:10.1097/PSY.0000000000000186.
107. Peng, T.K. Construct Validation of the Bem Sex Role Inventory in Taiwan. *Sex Roles* **2006**, *55*, 843–851, doi:10.1007/s11199-006-9136-6.
108. Peralta, R.L.; Steele, J.L.; Nofziger, S.; Rickles, M. The Impact of Gender on Binge Drinking Behavior Among U.S. College Students Attending a Midwestern University: An Analysis of Two Gender Measures. *Feminist Criminology* **2010**, *5*, 355–379, doi:10.1177/1557085110386363.
109. Raparelli, V.; Romiti, G.; Spugnardi, V.; Borgi, M.; Cangemi, R.; Basili, S.; Proietti, M.; the EVA Collaborative Group Gender-Related Determinants of Adherence to the Mediterranean Diet in Adults with Ischemic Heart Disease. *Nutrients* **2020**, *12*, 759, doi:10.3390/nu12030759.
110. Reynolds, G.L.; Fisher, D.G.; Dyo, M.; Huckabay, L.M. Using the Bem and Klein Grid Scores to Predict Health Services Usage by Men. *Behavioral Medicine* **2016**, *42*, 143–149, doi:10.1080/08964289.2016.1154003.
111. Ritter, D. Gender Role Orientation and Performance on Stereotypically Feminine and Masculine Cognitive Tasks. *Sex Roles* **2004**, *50*, 583–591, doi:10.1023/B:SERS.0000023077.91248.f7.
112. Russell, C.J.; Keel, P.K. Homosexuality as a Specific Risk Factor for Eating Disorders in Men. *Int. J. Eat. Disord.* **2002**, *31*, 300–306, doi:10.1002/eat.10036.
113. Sajatovic, M.; Micula-Gondek, W.; Tatsuoka, C.; Bialko, C. The Relationship of Gender and Gender Identity to Treatment Adherence Among Individuals With Bipolar Disorder. *Gender Medicine* **2011**, *8*, 261–268, doi:10.1016/j.genm.2011.06.002.
114. Stafford, L.; Dainton, M.; Haas, S. Measuring Routine and Strategic Relational Maintenance: Scale Revision, Sex versus Gender Roles, and the Prediction of Relational Characteristics. *Communication Monographs* **2000**, *67*, 306–323, doi:10.1080/03637750009376512.
115. Tardif, M.; Van Gijseghem, H. The Gender Identity of Pedophiles: What Does the Outcome Data Tell Us? *Journal of Child Sexual Abuse* **2005**, *14*, 57–74, doi:10.1300/J070v14n01_04.
116. Tate, C.C.; Ledbetter, J.N.; Youssef, C.P. A Two-Question Method for Assessing Gender Categories in the Social and Medical Sciences. *Journal of Sex Research* **2013**, *50*, 767–776, doi:10.1080/00224499.2012.690110.
117. Tate, C. The "Problem of Number" Revisited: The Relative Contributions of Psychosocial, Experiential, and Evolutionary Factors to the Desired Number of Sexual Partners. *Sex Roles* **2011**, *64*, 644–657, doi:10.1007/s11199-010-9774-6.
118. Vafaei, A.; Alvarado, B.; Tomás, C.; Muro, C.; Martinez, B.; Zunzunegui, M.V. The Validity of the 12-Item Bem Sex Role Inventory in Older Spanish Population: An Examination of the Androgyny Model. *Archives of Gerontology and Geriatrics* **2014**, *59*, 257–263, doi:10.1016/j.archger.2014.05.012.
119. Vafaei, A.; Ahmed, T.; Freire, A. do N.F.; Zunzunegui, M.V.; Guerra, R.O. Depression, Sex and Gender Roles in Older Adult Populations: The International Mobility in Aging Study (IMIAS). *PLoS ONE* **2016**, *11*, e0146867, doi:10.1371/journal.pone.0146867.
120. van Well, S.; Kolk, A.M.; Arrindell, W.A. Cross-Cultural Validity of the Masculine and Feminine Gender Role Stress Scales. *Journal of Personality Assessment* **2005**, *84*, 271–278, doi:10.1207/s15327752jpa8403_06.
121. Zhang, J.; Norvilitis, J.M.; Jin, S. Measuring Gender Orientation With the Bem Sex Role Inventory in Chinese Culture. *Sex Roles* **2001**, *44*, 237–251, doi:10.1023/A:1010911305338.
122. Taher, N.S. SELF-CONCEPT AND MASCULINITY/FEMININITY AMONG NORMAL MALE INDIVIDUALS AND MALES WITH GENDER IDENTITY DISORDER. *soc behav pers* **2007**, *35*, 469–478, doi:10.2224/sbp.2007.35.4.469.

123. Alavi, K.; Eftekhar, M.; Jalali Nadoushan, A.H. Comparison of Masculine and Feminine Gender Roles in Iranian Patients with Gender Identity Disorder. *Sexual Medicine* **2015**, *3*, 261–268, doi:10.1002/sm2.79.
124. Park, H.Y.; Jang, Y.E.; Oh, S.; Lee, P.B. Psychological Characteristics in Patients with Chronic Complex Regional Pain Syndrome: Comparisons with Patients with Major Depressive Disorder and Other Types of Chronic Pain. *JPR* **2020**, *Volume 13*, 389–398, doi:10.2147/JPR.S230394.
125. Woo, M.; Oei, T.P.S. The MMPI-2 Gender-Masculine and Gender-Feminine Scales: Gender Roles as Predictors of Psychological Health in Clinical Patients. *International Journal of Psychology* **2006**, *41*, 413–422, doi:10.1080/00207590500412185.
126. Woo, M.; Oei, T.P.S. Empirical Investigations of the MMPI-2 Gender-Masculine and Gender-Feminine Scales 1Dr Matthew Woo, Principal Psychologist, Is Now at the Department of Psychology, Institute of Mental Health, 10 Buangkok View, Singapore 539 747. *Journal of Individual Differences* **2008**, *29*, 1–10, doi:10.1027/1614-0001.29.1.1.
127. Langelier, D.M.; Cormie, P.; Bridel, W.; Grant, C.; Albinati, N.; Shank, J.; Daun, J.T.; Fung, T.S.; Davey, C.; Culos-Reed, S.N. Perceptions of Masculinity and Body Image in Men with Prostate Cancer: The Role of Exercise. *Support Care Cancer* **2018**, *26*, 3379–3388, doi:10.1007/s00520-018-4178-1.
128. Luyt, R. Masculinities Representations Inventory (MRI, English Version): A Measure of Gender (Re)Presentation. *The Journal of Men's Studies* **2018**, *26*, 157–183, doi:10.1177/1060826517736781.
129. Kowalczyk, R.; Skrzypulec, V.; Lew-Starowicz, Z.; Nowosielski, K.; Grabski, B.; Merk, W. Psychological Gender of Patients with Polycystic Ovary Syndrome: Gender Issues in PCOS. *Acta Obstetricia et Gynecologica Scandinavica* **2012**, *91*, 710–714, doi:10.1111/j.1600-0412.2012.01408.x.
130. Mandal, E.; Kocur, D. Psychological Masculinity, Femininity and Tactics of Manipulation in Patients with Borderline Personality Disorder. *Archives of Psychiatry and Psychotherapy* **2013**, *15*, 45–53.
131. McCreary, D.R.; Rhodes, N.D.; Saucier, D.M. [No Title Found]. *Sex Roles* **2002**, *47*, 169–177, doi:10.1023/A:1021099106603.
132. Rydberg Sterner, T.; Gudmundsson, P.; Seidu, N.; Bäckman, K.; Skoog, I.; Falk, H. A Psychometric Evaluation of a Swedish Version of the Positive–Negative Sex-Role Inventory (PN-SRI). *Societies* **2018**, *8*, 13, doi:10.3390/soc8010013.
133. Hirokawa, K.; Yamada, F.; Dohi, I.; Miyata, Y. EFFECT OF GENDER-TYPES ON INTERPERSONAL STRESS MEASURED BY BLINK RATE AND QUESTIONNAIRES: FOCUSING ON STEREOTYPICALLY SEX-TYPED AND ANDROGYNOUS TYPES. *soc behav pers* **2001**, *29*, 375–384, doi:10.2224/sbp.2001.29.4.375.
134. Weaver, J.; Sargent, S.L. Gender Role Inventory: In *Handbook of Research on Electronic Surveys and Measurements*; Reynolds, R.A., Woods, R., Baker, J.D., Eds.; IGI Global, 2007; pp. 367–370 ISBN 978-1-59140-792-8.
135. Ward, C.A. Models and Measurements of Psychological Androgyny: A Cross-Cultural Extension of Theory and Research. *Sex Roles* **2000**, *43*, 529–552, doi:10.1023/A:1007171500798.
136. Basu, J. Development of the Indian Gender Role Identity Scale. *Journal of the Indian Academy of Applied Psychology* **2010**, *36*, 25–34.
137. Basu, J.; DASGUPTA, S.; CHAKRABORTY, U.; Basu, S. Variants of the Indian Gender Role Identity Scale (IGRIS) for Different Age Groups in Bengali Population. *Journal of the Indian Academy of Applied Psychology* **2012**, *38*, 305–310.
138. Eisler, R.M.; Skidmore, J.R. Masculine Gender Role Stress: Scale Development and Component Factors in the Appraisal of Stressful Situations. *Behav Modif* **1987**, *11*, 123–136, doi:10.1177/01454455870112001.
139. Arrindell, W.A.; Kolk, A.M.; Martín, K.; Kwee, M.G.T.; Booms, E.O.H. Masculine Gender Role Stress: A Potential Predictor of Phobic and Obsessive–Compulsive Behaviour. *Journal of Behavior Therapy and Experimental Psychiatry* **2003**, *34*, 251–267, doi:10.1016/j.jbtep.2003.10.002.
140. Kaźmierczak, M.; Pastwa-Wojciechowska, B.; Błażek, M. A Multidimensional Model of Empathy, and the Occurrence of Personality Disorders and Stress in Social Settings. *Acta Neuropsychologica* **2013**, *11*, 113–125.
141. Mussap, A.J. Masculine Gender Role Stress and the Pursuit of Muscularity. *International Journal of Men's Health* **2008**, *7*, 72–89, doi:10.3149/jmh.0701.72.
142. Susánszky, A.; Döbrössy, B. The Correlation Between Body Mass Index and Gender Role Stress Among Young Hungarian Males. *EJMH* **2019**, *14*, 190–202, doi:10.5708/EJMH.14.2019.1.11.
143. Swartout, K.M.; Parrott, D.J.; Cohn, A.M.; Hagman, B.T.; Gallagher, K.E. Development of the Abbreviated Masculine Gender Role Stress Scale. *Psychological Assessment* **2015**, *27*, 489–500, doi:10.1037/a0038443.
144. Gillespie, B.L.; Eisler, R.M. Development of the Feminine Gender Role Stress Scale: A Cognitive-Behavioral Measure of Stress, Appraisal, and Coping for Women. *Behav Modif* **1992**, *16*, 426–438, doi:10.1177/01454455920163008.

145. Richmond, K.; Levant, R.; Smalley, B.; Cook, S. The Femininity Ideology Scale (FIS): Dimensions and Its Relationship to Anxiety and Feminine Gender Role Stress. *Women & Health* **2015**, *55*, 263–279, doi:10.1080/03630242.2014.996723.
146. Shea, M.; Wong, Y.J.; Wang, S.; Wang, S.; Jimenez, V.; Hickman, S.J.; LaFollette, J.R. Toward a Constructionist Perspective of Examining Femininity Experience: The Development and Psychometric Properties of the Subjective Femininity Stress Scale. *Psychology of Women Quarterly* **2014**, *38*, 275–291, doi:10.1177/0361684313509591.
147. O'Neil, JamesM.; Helms, BarbaraJ.; Gable, RobertK.; David, L.; Wrightsman, LawrenceS. Gender-Role Conflict Scale: College Men's Fear of Femininity. *Sex Roles* **1986**, *14*, doi:10.1007/BF00287583.
148. Hammer, J.H.; McDermott, R.C.; Levant, R.F.; McKelvey, D.K. Dimensionality, Reliability, and Validity of the Gender-Role Conflict Scale–Short Form (GRCS-SF). *Psychology of Men & Masculinity* **2018**, *19*, 570–583, doi:10.1037/men0000131.
149. Herdman, K.J.; Fuqua, D.R.; Choi, N.; Newman, J.L. Gender Role Conflict Scale: Validation for a Sample of Gay Men and Lesbian Women. *Psychol Rep* **2012**, *110*, 227–232, doi:10.2466/02.07.PR0.110.1.227-232.
150. Jones, K.D.; Heesacker, M. Addressing the Situation: Some Evidence for the Significance of Microcontexts with the Gender Role Conflict Construct. *Psychology of Men & Masculinity* **2012**, *13*, 294–307, doi:10.1037/a0025797.
151. Komlenac, N.; Siller, H.; Bliem, H.R.; Hochleitner, M. Validation of the Internal Structure of a German-Language Version of the Gender Role Conflict Scale – Short Form. *Front. Psychol.* **2018**, *9*, 1161, doi:10.3389/fpsyg.2018.01161.
152. Lease, S.H.; Çiftçi, A.; Demir, A.; Boyraz, G. Structural Validity of Turkish Versions of the Gender Role Conflict Scale and Male Role Norms Scale. *Psychology of Men & Masculinity* **2009**, *10*, 273–287, doi:10.1037/a0017044.
153. Moradi, B.; Tokar, D.M.; Schaub, M.; Jome, L.M.; Serna, G.S. Revisiting the Structural Validity of the Gender Role Conflict Scale. *Psychology of Men & Masculinity* **2000**, *1*, 62–69, doi:10.1037/1524-9220.1.1.62.
154. Norwalk, K.E.; Vandiver, B.J.; White, A.M.; Englar-Carlson, M. Factor Structure of the Gender Role Conflict Scale in African American and European American Men. *Psychology of Men & Masculinity* **2011**, *12*, 128–143, doi:10.1037/a0022799.
155. Wester, S.R.; Vogel, D.L.; O'Neil, J.M.; Danforth, L. Development and Evaluation of the Gender Role Conflict Scale Short Form (GRCS-SF). *Psychology of Men & Masculinity* **2012**, *13*, 199–210, doi:10.1037/a0025550.
156. Zhang, C.; Blashill, A.J.; Wester, S.R.; O'Neil, J.M.; Vogel, D.L.; Wei, J.; Zhang, J. Factor Structure of the Gender Role Conflict Scale-Short Form in Chinese Heterosexual and Gay Samples. *Psychology of Men & Masculinity* **2015**, *16*, 229–233, doi:10.1037/a0036154.
157. Mincey, K.; Alfonso, M.; Hackney, A.; Luque, J. Being a Black Man: Development of the Masculinity Inventory Scale (MIS) for Black Men. *The Journal of Men's Studies* **2014**, *22*, 167–179, doi:10.3149/jms.2203.167.
158. Brown, D.L.; Blackmon, S.; Rosnick, C.B.; Griffin-Fennell, F.D.; White-Johnson, R.L. Initial Development of a Gendered-Racial Socialization Scale for African American College Women. *Sex Roles* **2017**, *77*, 178–193, doi:10.1007/s11199-016-0707-x.
159. Clark, J.A.; Inui, T.S.; Silliman, R.A.; Bokhour, B.G.; Krasnow, S.H.; Robinson, R.A.; Spaulding, M.; Talcott, J.A. Patients' Perceptions of Quality of Life After Treatment for Early Prostate Cancer. *JCO* **2003**, *21*, 3777–3784, doi:10.1200/JCO.2003.02.115.
160. Kozee, H.B.; Tylka, T.L.; Bauerband, L.A. Measuring Transgender Individuals' Comfort With Gender Identity and Appearance: Development and Validation of the Transgender Congruence Scale. *Psychology of Women Quarterly* **2012**, *36*, 179–196, doi:10.1177/0361684312442161.
161. Barr, S.M.; Budge, S.L.; Adelson, J.L. Transgender Community Belongingness as a Mediator between Strength of Transgender Identity and Well-Being. *Journal of Counseling Psychology* **2016**, *63*, 87–97, doi:10.1037/cou0000127.
162. Egan, S.K.; Perry, D.G. Gender Identity: A Multidimensional Analysis with Implications for Psychosocial Adjustment. *Developmental Psychology* **2001**, *37*, 451–463, doi:10.1037/0012-1649.37.4.451.
163. Reisen, C.A.; Brooks, K.D.; Zea, M.C.; Poppen, P.J.; Bianchi, F.T. Can Additive Measures Add to an Intersectional Understanding? Experiences of Gay and Ethnic Discrimination among HIV-Positive Latino Gay Men. *Cultural Diversity and Ethnic Minority Psychology* **2013**, *19*, 208–217, doi:10.1037/a0031906.
164. Docter, R.F.; Fleming, J.S. Measures of Transgender Behavior. *Archives of Sexual Behavior* **2001**, *30*, 255–271, doi:10.1023/A:1002795929547.

165. Bockting, W.O.; Miner, M.H.; Swinburne Romine, R.E.; Dolezal, C.; Robinson, B. "Bean" E.; Rosser, B.R.S.; Coleman, E. The Transgender Identity Survey: A Measure of Internalized Transphobia. *LGBT Health* **2020**, *7*, 15–27, doi:10.1089/lgbt.2018.0265.
166. Storms, M.D. Sex Role Identity and Its Relationships to Sex Role Attributes and Sex Role Stereotypes. *Journal of Personality and Social Psychology* **1979**, *37*, 1779–1789, doi:10.1037/0022-3514.37.10.1779.
167. Bejerot, S.; Eriksson, J.M. Sexuality and Gender Role in Autism Spectrum Disorder: A Case Control Study. *PLoS ONE* **2014**, *9*, e87961, doi:10.1371/journal.pone.0087961.
168. Wade, J.C.; Gelso, C.J. Reference Group Identity Dependence Scale: A Measure of Male Identity. *The Counseling Psychologist* **1998**, *26*, 384–412, doi:10.1177/0011000098263002.
169. Cicero, E.C.; Reisner, S.L.; Merwin, E.I.; Humphreys, J.C.; Silva, S.G. Application of Behavioral Risk Factor Surveillance System Sampling Weights to Transgender Health Measurement. *Nursing Research* **2020**, *69*, 307–315, doi:10.1097/NNR.0000000000000428.
170. Bauerband, L.A.; Teti, M.; Velicer, W.F. Measuring Minority Stress: Invariance of a Discrimination and Vigilance Scale across Transgender and Cisgender LGBQ Individuals. *Psychology & Sexuality* **2019**, *10*, 17–30, doi:10.1080/19419899.2018.1520143.
171. Bazzi, A.R.; Whorms, D.S.; King, D.S.; Potter, J. Adherence to Mammography Screening Guidelines Among Transgender Persons and Sexual Minority Women. *Am J Public Health* **2015**, *105*, 2356–2358, doi:10.2105/AJPH.2015.302851.
172. Christian, R.; Mellies, A.A.; Bui, A.G.; Lee, R.; Kattari, L.; Gray, C. Measuring the Health of an Invisible Population: Lessons from the Colorado Transgender Health Survey. *J GEN INTERN MED* **2018**, *33*, 1654–1660, doi:10.1007/s11606-018-4450-6.
173. Fraser, G.; Bulbulia, J.; Greaves, L.M.; Wilson, M.S.; Sibley, C.G. Coding Responses to an Open-Ended Gender Measure in a New Zealand National Sample. *The Journal of Sex Research* **2020**, *57*, 979–986, doi:10.1080/00224499.2019.1687640.
174. Ewalds-Kvist, S.B.M.; Rantala, M.; Nikkanen, V.; Selander, R.K.; Lertola, K. The Response of the Finnish Man to Vasectomy. *Psychology, Health & Medicine* **2003**, *8*, 355–369, doi:10.1080/1354850031000135795.
175. Ballering, A.V.; Bonvanie, I.J.; Olde Hartman, T.C.; Monden, R.; Rosmalen, J.G.M. Gender and Sex Independently Associate with Common Somatic Symptoms and Lifetime Prevalence of Chronic Disease. *Social Science & Medicine* **2020**, *253*, 112968, doi:10.1016/j.socscimed.2020.112968.
176. Tanner, J.M. CURRENT ADVANCES IN THE STUDY OF PHYSIQUE. *The Lancet* **1951**, *257*, 574–579, doi:10.1016/S0140-6736(51)92260-X.
177. Dubey, A. IMPORTANCE OF ANDROGYNY SCORE IN DIFFERENTIATION BETWEEN SEXES AND AS A DIAGNOSTIC TOOL IN SCHIZOPHRENIC FEMALES. *jemds* **2015**, *04*, 1765–1768, doi:10.14260/jemds/2015/252.
178. Mishra, V.; Sharma, S.; Kulsreshtha, V.; Kumar, V.; Singh, D. Anthropometric Study Revealed: Androgyny Score as an Important Tool in Early Diagnosis of Schizophrenia. *Journal of Anatomical Society of India* **2011**, *60*, 207–209, doi:10.1016/S0003-2778(11)80028-6.
179. The GenIUSS Group. *Best Practices for Asking Questions to Identify Transgender and Other Gender Minority Respondents on Population-Based Surveys.*; The Williams Institute: Los Angeles, CA, 2014;
180. Smith, Y.L.S.; van Goozen, S.H.M.; Kuiper, A.J.; Cohen-Kettenis, P.T. Transsexual Subtypes: Clinical and Theoretical Significance. *Psychiatry Research* **2005**, *137*, 151–160, doi:10.1016/j.psychres.2005.01.008.
181. Suppakitjanusant, P.; Ji, Y.; Stevenson, M.O.; Chantrapanichkul, P.; Sineath, R.C.; Goodman, M.; Alvarez, J.A.; Tangpricha, V. Effects of Gender Affirming Hormone Therapy on Body Mass Index in Transgender Individuals: A Longitudinal Cohort Study. *Journal of Clinical & Translational Endocrinology* **2020**, *21*, 100230, doi:10.1016/j.jcte.2020.100230.
182. Lacasse, A.; Pagé, M.G.; Choinière, M.; Dorais, M.; Vissandjée, B.; Nguefack, H.L.N.; Katz, J.; Samb, O.M.; Vanasse, A.; on behalf of the TORSADE Cohort Working Group Conducting Gender-Based Analysis of Existing Databases When Self-Reported Gender Data Are Unavailable: The GENDER Index in a Working Population. *Can J Public Health* **2020**, *111*, 155–168, doi:10.17269/s41997-019-00277-2.
183. Smith, P.M.; Koehoorn, M. Measuring Gender When You Don't Have a Gender Measure: Constructing a Gender Index Using Survey Data. *Int J Equity Health* **2016**, *15*, 82, doi:10.1186/s12939-016-0370-4.

Supplementary Figure S1: Number of Applications

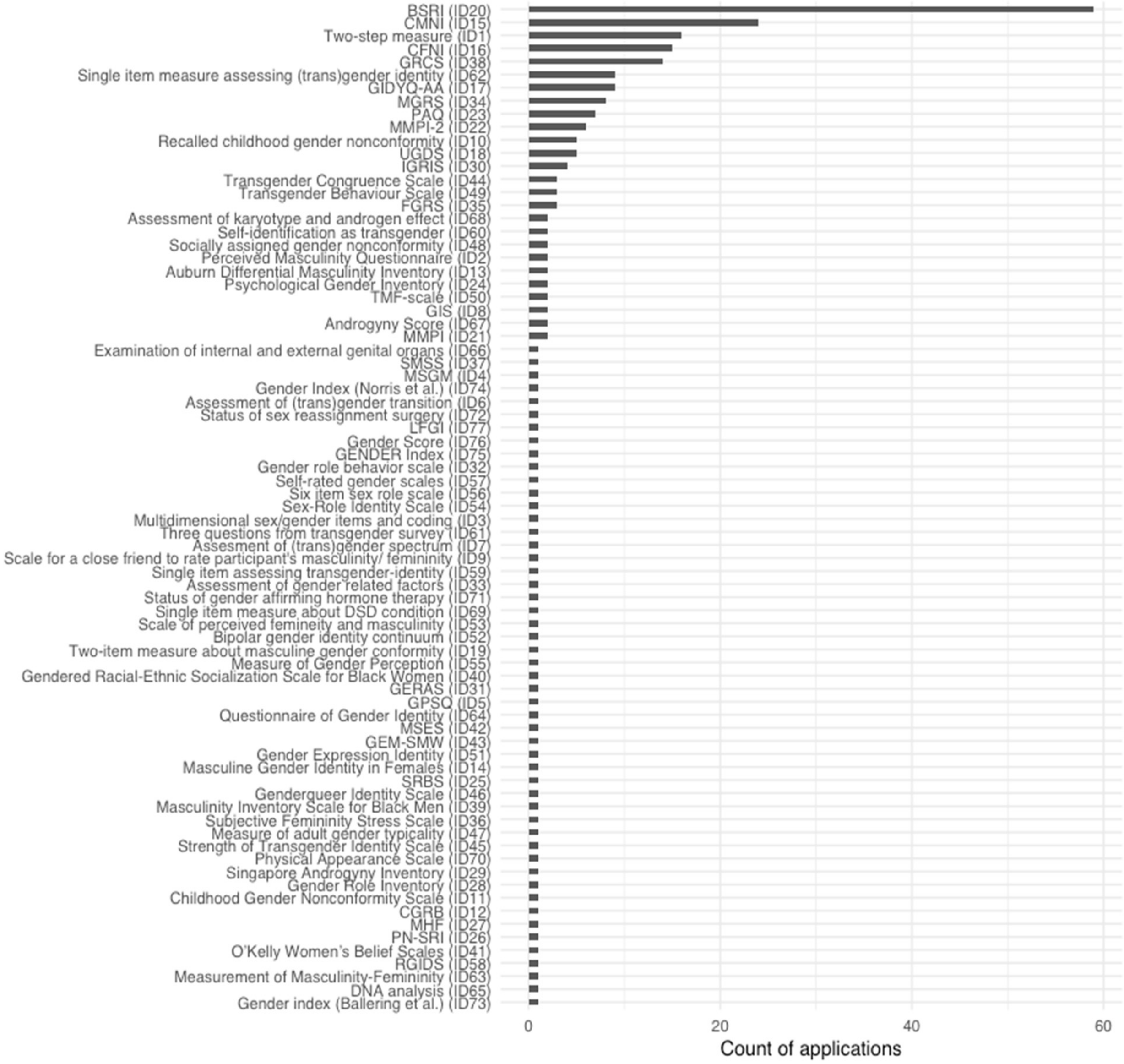

Figure S1: Number of Applications per Tool (N = 77)

Supplementary Figure S2: Population size

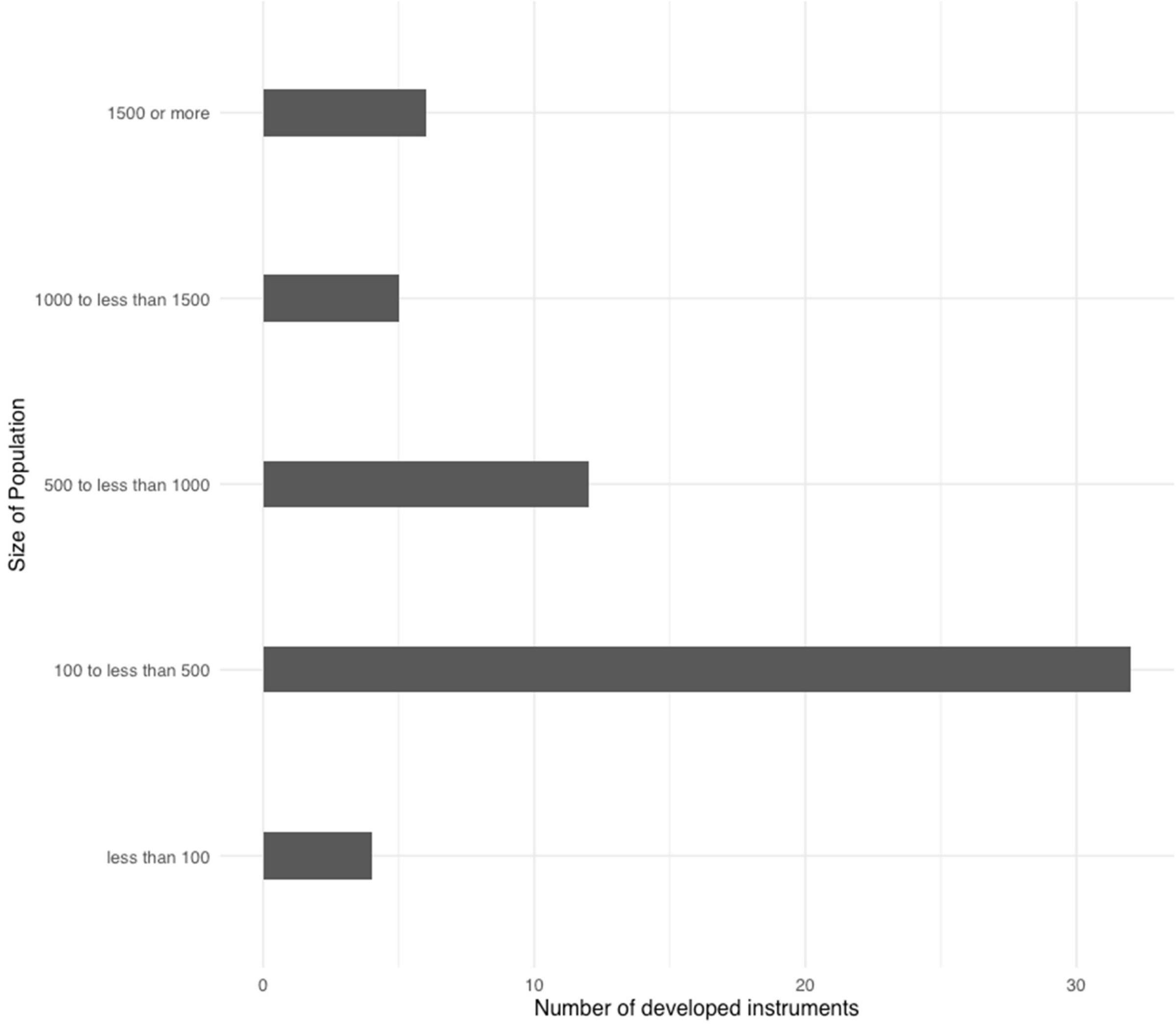

Figure S2: Size of population the instruments were developed with (n = 59)

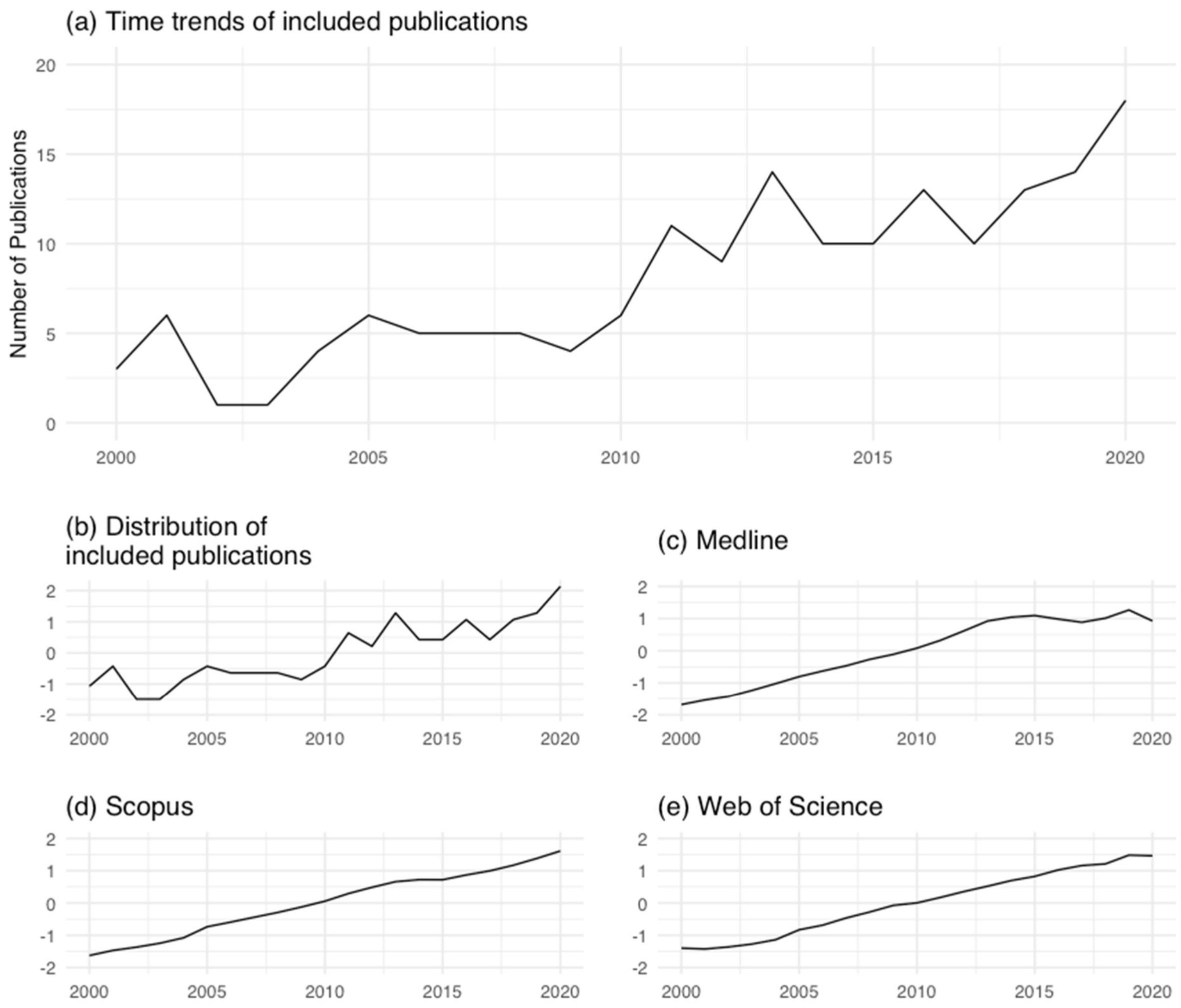

Supplementary Figure S3: Temporal trends of the included publications

Figure S3: a) Time Trend of included publications from January 2000 to August 2020. b) Standardised distribution of the number of publications included within this review from January 2000 to August 2020. c) Standardised distribution of the number of publications contained in Medline (via Ovid) from January 2000 to August 2020. c) Standardised distribution of the number of publications contained Scopus from January 2000 to August 2020. d) Standardised distribution of the number of publications contained in Web of Science from January 2000 to August 2020.

Supplementary Table S4: Categorisation of the tools according to their underlying concepts of sex and/or gender

ID	Instrument
**(I) Gender, sex or sex and/or gender is recorded as male/masculine or female/feminine only**	
**(I.1) Instruments to define the belonging to a certain sex and/or gender – either self-definition or by others**	
**(I.1.1) Sex/gender is conceptualised as distinct**	
ID21	Minnesota Multiphasic Personality Inventory (MMPI)
ID52	Bipolar gender identity continuum
ID53	Scale of perceived femininity and masculinity
ID73	Gender Index
ID74	Gender Index
ID75	GENDER Index
ID76	Gender Score
ID77	Labour Force Gender Index (LFGI)
**(I.1.2) Sex and/or gender is conceptualised as multiple**	
ID9	Scale for a close friend to rate participant's masculinity/ femininity
ID23	Personal Attributes Questionnaire (PAQ)
ID25	Sex Role Behaviour Scale (SRBS)
ID31	Gender-Related Attributes Survey
ID32	Gender role behaviour scale
ID50	Traditional Masculinity-Femininity (TMF) scale
ID54	Sex-Role Identity Scale (SRIS)
ID56	Six item sex role scale
ID57	Self-rated gender scales
ID70	Physical Appearance Scale
**(I.1.2.1) Derivation of further categories such as androgyne or indifferent**	
ID20	Bem Sex Role Inventory (BSRI)
ID22	Minnesota Multiphasic Personality Inventory 2 (MMPI-2)
ID24	Psychological Gender Inventory
ID26	Positive–Negative Sex-Role Inventory (PN-SRI)
ID27	Masculinity - Humanity - Femininity (MHF) scale
ID29	Singapore Androgyny Inventory
ID30	Indian Gender Role Identity Scale (IGRIS)
ID67	Androgyny Score

(I.2) Instruments to measure conformity, grades of belonging or consequences of belonging to a certain sex and/or gender

(I.2.1) Level of belonging/conformity

ID2	Perceived Masculinity Questionnaire
ID10	Recalled childhood gender nonconformity
ID11	Childhood Gender Nonconformity Scale
ID12	Childhood gender role behaviour (CGRB)
ID13	Auburn Differential Masculinity Inventory
ID14	Masculine Gender Identity in Females
ID15	Conformity to Masculine Norms Inventory (CMNI)
ID16	Conformity to Feminine Norms Inventory (CFNI)
ID19	Socially assigned gender nonconformity
ID39	Masculinity Inventory Scale (MIS) for Black Men
ID43	Gender Expression Measure among Sexual Minority Women (GEM-SMW)
ID47	Measure of adult gender typicality
ID48	Gender nonconformity in self-presentation (of gay men)
ID58	Reference Group Identity Dependence Scale (RGIDS)

(I.2.2) Consequences of the belonging to a certain gender

ID34	Masculine Gender Role Stress
ID35	Feminine Gender Role Stress
ID36	Subjective Femininity Stress Scale
ID37	Subjective Masculinity Stress Scale (SMSS)
ID38	Gender Role Conflict Scale (GRCS)
ID40	Gendered Racial-Ethnic Socialization Scale for Black Women
ID41	O'Kelly Women's Belief Scales
ID42	Masculine Self-esteem Scale (MSES)

II. Gender, sex or sex and/or gender is recorded in further/other categories than male/masculine or female/feminine

(II.1) Instruments to define the belonging to a certain sex and/or gender – either self-definition or by others

ID1	Two-step measures
ID3	Multidimensional sex/gender items
ID4	Multidimensional Sex/Gender Measure (MSGM)
ID8	Gender Identity Scale (GIS)
ID17	Gender Identity/Gender Dysphoria Questionnaire for Adolescents and Adults (GIGDQ-AA)
ID18	Utrecht Gender Dysphoria Scale (UGDS)

ID49	Transgender Behaviour Scale
ID59	Single item assessing transgender-identity
ID60	Self-identification as transgender
ID61	Three questions from transgender survey
ID62	Single item measure assessing gender identity
ID65	DNA analysis
ID66	Examination of internal and external genital organs
ID68	Assessment of karyotype and androgen effect
ID69	Single item measure about DSD condition

(II.2) Instruments to measure conformity or grade of belonging to a certain sex and/or gender

ID5	Gender Preoccupation and Stability Questionnaire (GPSQ)
ID6	Assessment of (trans)gender transition
ID7	Assessment of (trans)gender spectrum
ID44	Transgender Congruence Scale
ID45	Strength of Transgender Identity Scale
ID51	Gender Expression Identity
ID71	Status of gender affirming hormone therapy
ID72	Sex reassignment surgery

(II.3) Mixed format – instruments that define the belonging to a sex and/or gender category and measure the grade of belonging

ID46	Genderqueer Identity (GQI) Scale
ID55	Measure of Gender Perception

"unclear"

ID28	Gender role inventory
ID33	Assessment of gender related factors

Since no further information are available the instruments ID63 and ID64 are excluded from this categorisation.

Supplementary Figure S4: Consideration of further social categories

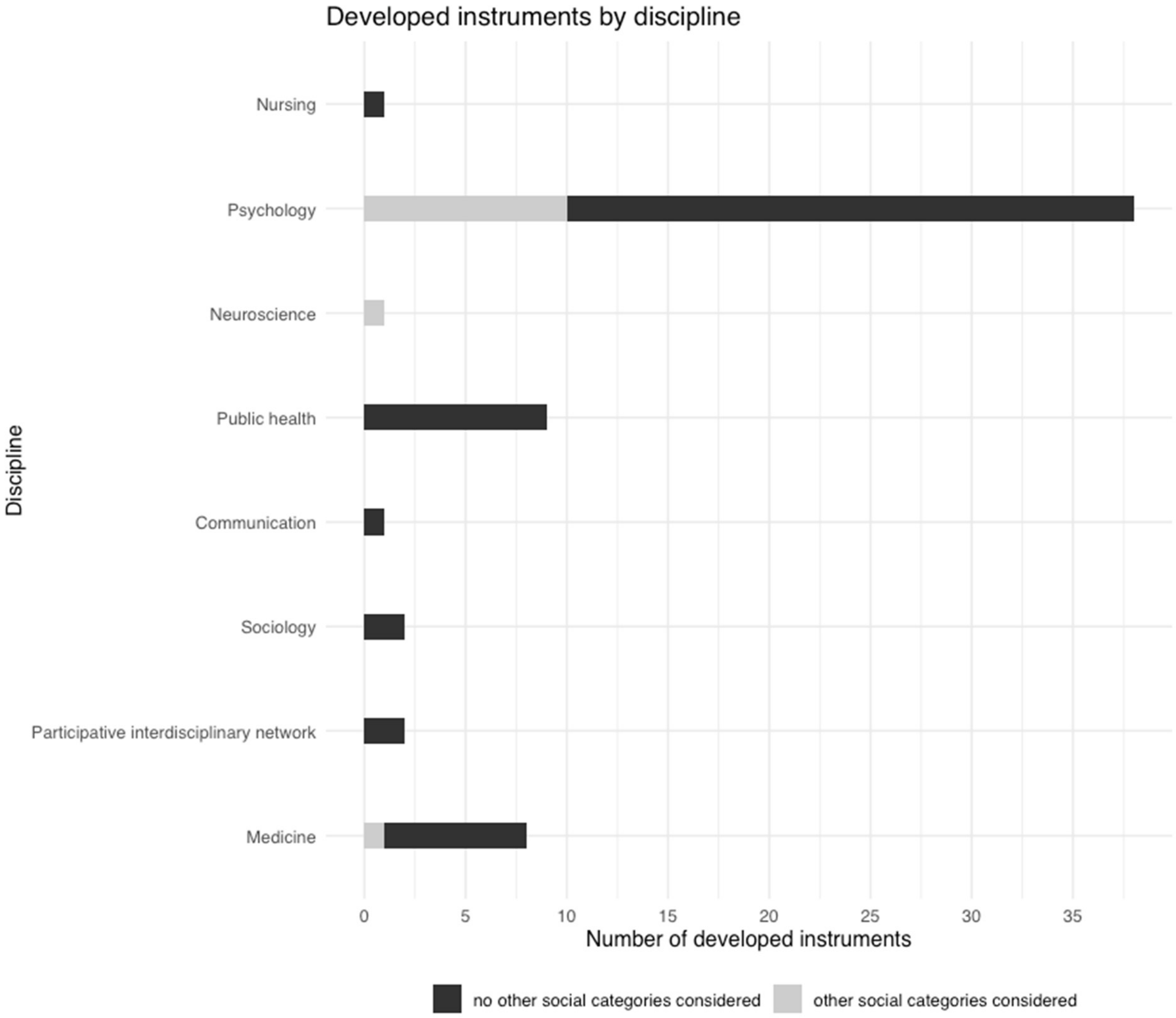

Figure S4: Number of instruments developed that take other social categories into account, by discipline (N = 62)
